# Activation of serum/glucocorticoid-induced kinase 1 (SGK1) is important to maintain skeletal muscle homeostasis and prevent atrophy

**DOI:** 10.1002/emmm.201201443

**Published:** 2012-11-19

**Authors:** Eva Andres-Mateos, Heinrich Brinkmeier, Tyesha N Burks, Rebeca Mejias, Daniel C Files, Martin Steinberger, Arshia Soleimani, Ruth Marx, Jessica L Simmers, Benjamin Lin, Erika Finanger Hedderick, Tom G Marr, Brian M Lin, Christophe Hourdé, Leslie A Leinwand, Dietmar Kuhl, Michael Föller, Silke Vogelsang, Ivan Hernandez-Diaz, Dana K Vaughan, Diego Alvarez de la Rosa, Florian Lang, Ronald D Cohn

**Affiliations:** 1 McKusick-Nathans Institute of Genetic Medicine, Johns Hopkins University School of MedicineBaltimore, MD, USA; 2 Institute of Pathophysiology, Ernst Moritz Arndt University of GreifswaldKarlsburg, Germany; 3 Department of Neurology, Johns Hopkins University School of MedicineBaltimore, MD, USA; 4 Hiberna CorporationBoulder, CO, USA; 5 Institut de Myologie; CNRS UMR 7215; INSERM, UMR_S 974; Université Pierre et Marie Curie-Paris6Paris, France; 6 Department of Molecular, Cellular, and Developmental Biology, University of ColoradoBoulder, CO, USA; 7 University Medical Center Hamburg-Eppendorf (UKE)Hamburg, Germany; 8 Department of Physiology, University of TübingenTübingen, Germany; 9 Department of Neuropathology, Ernst Moritz Arndt University of GreifswaldKarlsburg, Germany; 10 Department of Physiology and Institute of Biomedical Technologies, University of La LagunaTenerife, Spain; 11 Department of Biology and Microbiology, University of Wisconsin OshkoshOshkosh, WI, USA; 12 Department of Pediatrics, Johns Hopkins University School of MedicineBaltimore, MD, USA

**Keywords:** hibernation, muscle atrophy, muscle homeostasis, muscle hypertrophy, SGK1

## Abstract

Maintaining skeletal muscle mass is essential for general health and prevention of disease progression in various neuromuscular conditions. Currently, no treatments are available to prevent progressive loss of muscle mass in any of these conditions. Hibernating mammals are protected from muscle atrophy despite prolonged periods of immobilization and starvation. Here, we describe a mechanism underlying muscle preservation and translate it to non-hibernating mammals. Although Akt has an established role in skeletal muscle homeostasis, we find that serum- and glucocorticoid-inducible kinase 1 (SGK1) regulates muscle mass maintenance via downregulation of proteolysis and autophagy as well as increased protein synthesis during hibernation. We demonstrate that SGK1 is critical for the maintenance of skeletal muscle homeostasis and function in non-hibernating mammals in normal and atrophic conditions such as starvation and immobilization. Our results identify a novel therapeutic target to combat loss of skeletal muscle mass associated with muscle degeneration and atrophy.

## INTRODUCTION

Skeletal muscle is the largest organ in the human body, comprising approximately 50% of total body weight. Maintenance of muscle mass and physiology is essential for general health. Disuse (*e.g.* immobilization, denervation and microgravity), inherited neuromuscular disorders and aging all result in debilitating loss of skeletal muscle (Saini et al, 2009). Loss of skeletal muscle mass not only increases morbidity and mortality, but also increases the incidence of pathologic fractures, functional deterioration and institutionalization (Degens & Alway, 2006). Despite decades of research, no treatments have been characterized to prevent loss of muscle mass in inherited and/or acquired forms of neuromuscular conditions.

Muscle mass preservation results from maintaining a homeostatic balance of protein synthesis and degradation. Understanding the mechanisms underlying the preservation of skeletal muscle tissue is critical for the development of therapeutic strategies to combat loss of muscle mass. This study takes an innovative approach to address this question in a model organism that has innate protective mechanisms against muscle loss: a hibernating rodent. We analysed the 13-lined ground squirrel (*Ictidomys tridecemlineatus*), a naturally occurring, hibernating animal (Vaughan et al, 2006). These rodents spend approximately half of the year in hibernation. Bradycardia, hypothermia, episodic ventilation, lack of food and water intake, and immobility characterize the torpor state. While the molecular mechanisms determining the hibernator's marked resilience against atrophy have not been elucidated, changes in mammalian target of rapamycin (mTOR) signalling as well as epigenetically mediated pathways have been suggested to play an important role (Lee et al, 2010; Morin & Storey, 2009; Nowell et al, 2011; Shavlakadze & Grounds, 2006).

In non-hibernating mammals, current paradigm dictates that muscle cell strength, growth and survival are dependent upon the PI3K/Akt/mTOR pathway, which is activated in response to several growth factors including insulin-growth factor-I (IGF-1; Glass, 2003). The binding of IGF-1 to its receptor triggers the activation of phosphatidylinositol 3-kinase (PI3K) and the translocation of the serine/threonine kinase Akt (also called Akt1 and PKB for protein kinase B) to the membrane. This process facilitates the phosphorylation and activation of Akt at serine-478 (S478) by the 3-phosphoinositide-dependent kinase 1 (PDK-1). Akt is a vital mediator of several processes involved in skeletal muscle homeostasis and survival. Direct and indirect targets downstream of Akt include mTOR, p70^S6K^ and eukaryotic translation initiation factor 4E binding protein 1 (4E-BP1)—key regulatory proteins involved in translation and protein synthesis (Glass, 2003; Saini et al, 2009). In addition, Akt inactivates Foxo3a by phosphorylation at serine-253 (S253) and threonine-32 (T32; Brunet et al, 2001). Inactivation of Foxo3a leads to decreased expression of target genes mediating skeletal muscle atrophy, atrogin-1 and MuRF1 and autophagy (*e.g.* LC3B, beclin, ATG7; Mammucari et al, 2007; Zhao et al, 2007). Several studies in mammals have shown that this pathway is altered in skeletal muscle during conditions of disuse and starvation (Glass, 2010).

Serum- and glucocorticoid-induced kinase 1 (SGK1) belongs to a family of serine/threonine kinases that shares 45–55% similarity with Akt, cAMP-dependent protein kinase, p70^S6K^ and protein kinase C with respect to their catalytic domains (Webster et al, 1993). Akt primarily phosphorylates Foxo3a at serine-253, while SGK1 has a higher affinity for serine-315, and both Akt and SGK1 phosphorylate threonine-32 with similar affinity (Brunet et al, 2001). In this study, we show that SGK1 exhibits a previously unknown role in mediating skeletal muscle homeostasis and function in hibernating and non-hibernating mammals. SGK1 mediates protection by inhibition of Foxo3a-induced atrophy and autophagy and by the activation of mTOR signalling. We propose that therapeutic modulation of SGK1 may be beneficial in conditions associated with muscle atrophy or degeneration.

## RESULTS

### Skeletal muscle size and morphology are not altered during hibernation

**Figure 1:**
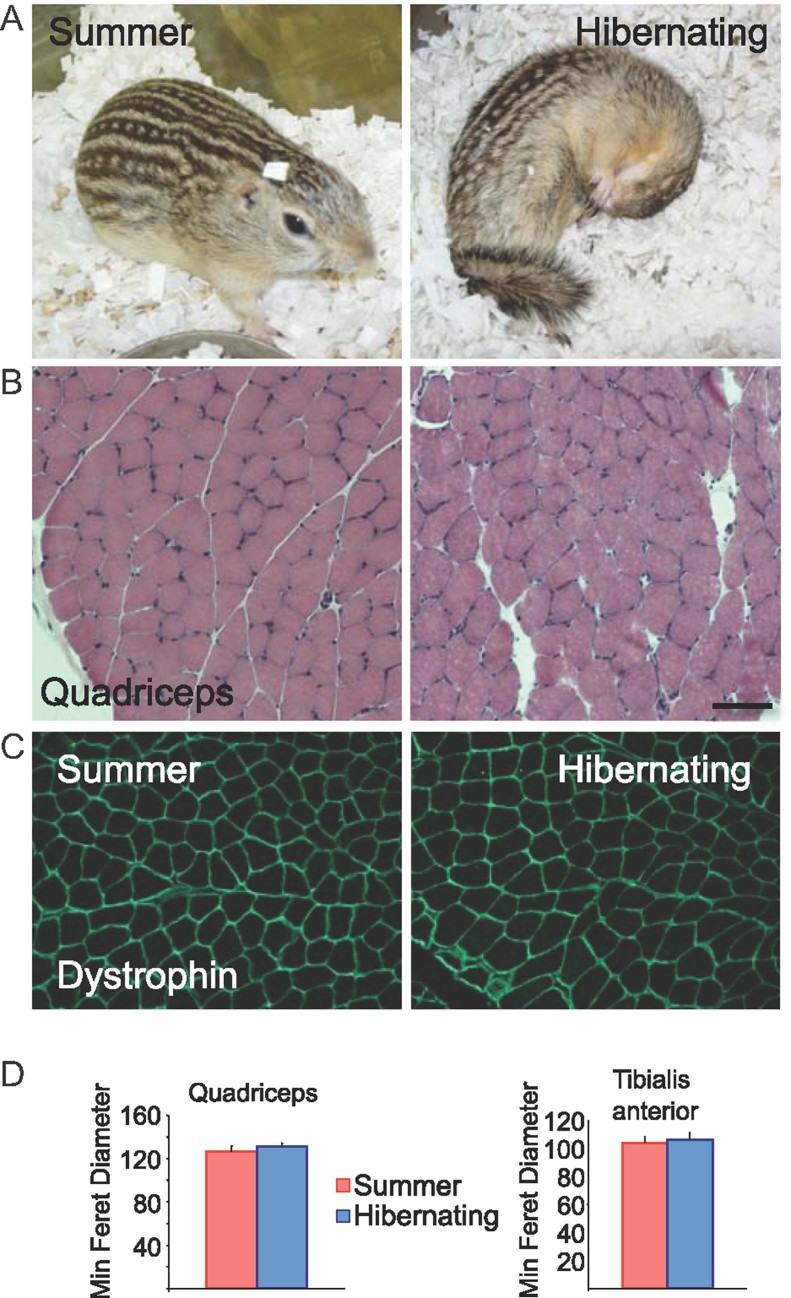
Normal skeletal muscle morphology in hibernating squirrels Left column, an active summer squirrel; right column, a torpid squirrel.The morphology of quadriceps is unchanged by hibernation as seen in haematoxylin and eosin (H&E) stained sections (scale bar 90 µm).Dystrophin staining was performed to outline the sarcolemma to determine percentage distribution of minimum Feret's diameter.Average ± SD of minimum Feret's diameter in quadriceps (*p* = 0.26) and tibialis anterior (*p* = 0.33) muscles is not significantly different between summer and hibernation.

Prolonged periods of immobilization and/or starvation cause significant muscle atrophy, defined by reduced muscle mass, muscle fiber size and muscle function, in various mammals including humans. Specifically, artificial limb immobilization in a mouse for 12–18 days causes a 45% loss of skeletal muscle mass, while mice deprived of food for 48 h lose approximately 15% muscle mass (Hudson & Franklin, 2002; Jagoe et al, 2002). Histological evaluation of quadriceps muscles collected from ground squirrels exposed to 6 months of immobility with no food or water intake and from active summer squirrels showed no morphological differences (Fig 1A and B). Muscles collected from the diaphragm, gastrocnemius and tibialis anterior (TA) also did not display variation in muscle architecture, composition or size between hibernating and summer squirrels. Supporting these observations, quantitative morphometric analysis of muscle fiber size revealed no significant changes in fiber size of quadriceps (composed of slow and fast muscle fibers) and TA muscles (mainly composed of fast muscle fibers) demonstrating preservation of muscle fiber size independently of fiber type composition (Fig 1C and D and Supporting Information Fig S1A). Despite extended periods of immobilization and starvation, which normally favour the development of muscle atrophy, the skeletal muscle mass, structure and morphometric values of the hibernating ground squirrel remain unchanged.

### Increased activation of mTOR and inactivation of Foxo3a are independent of Akt

**Figure 2:**
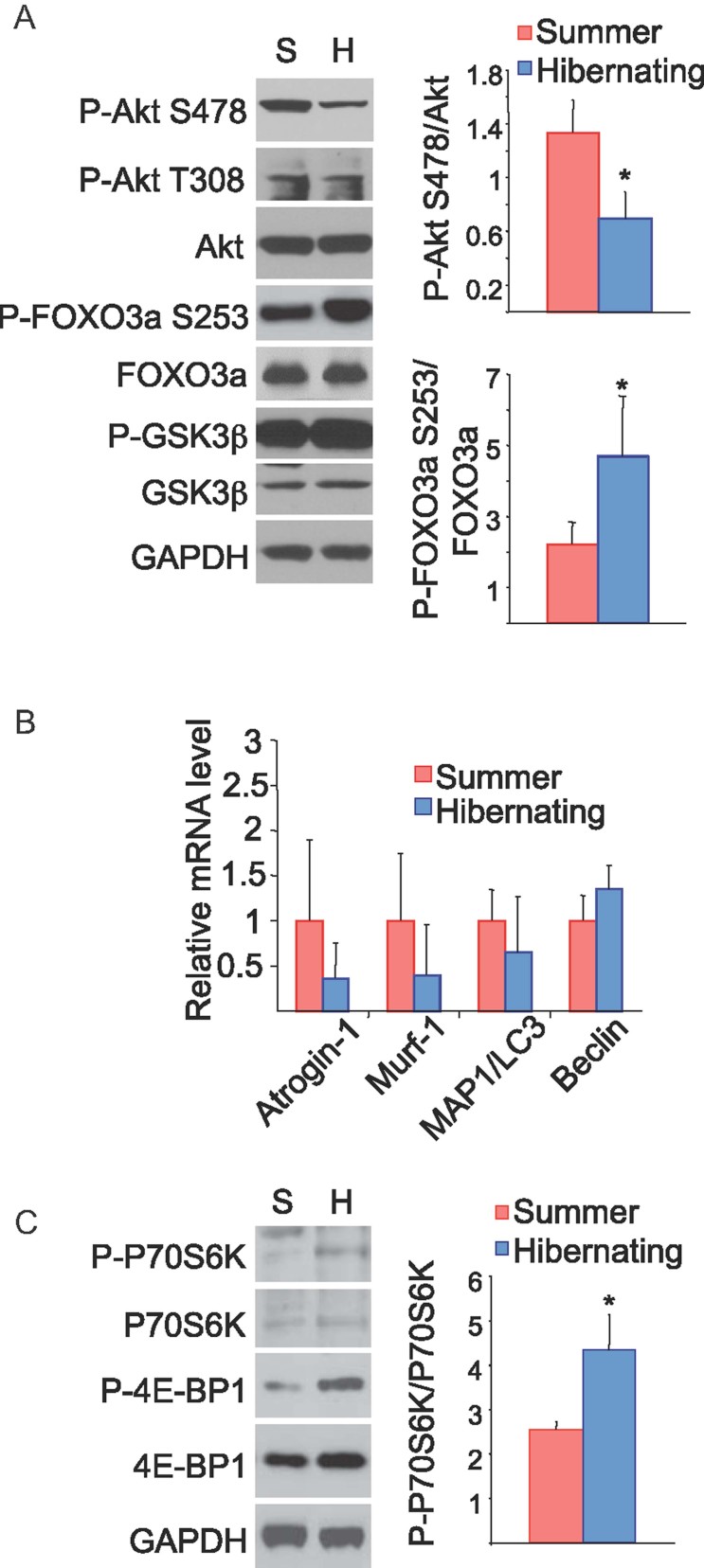
Evaluation of protein degradation and synthesis pathways Western blot of quadriceps muscle from summer active (S) and hibernating (H) squirrels using antibodies against the proteins indicated. An increased abundance of P-Foxo3a (serine-253) is accompanied by a decrease in P-Akt (serine-478). Corresponding densitometry of P-Akt and P-Foxo3a as a function of total Akt and Foxo3a.Relative mRNA levels for four Foxo3a downstream targets show no significant changes during hibernation.Western blots analysis of mTOR downstream targets shows significant upregulation of P-P70^S6K^ and P-4E-BP1 during hibernation.

The PI3K/Akt/mTOR pathway stimulates myofiber growth and protein synthesis and regulates protein degradation (Bodine et al, 2001). We assessed members of this pathway in skeletal muscle of hibernating and non-hibernating animals. Levels of phosphorylated (inactive) Foxo3a at serine-253 were increased (Fig 2A). Evaluation of downstream targets of Foxo3a by real-time PCR revealed no significant increase in expression of atrophy or autophagy genes including atrogin-1 and MuRF1 or MAP1/LC3B during hibernation (Fig 2B). Analysis of the proteasome during hibernation showed an elevation of ubiquitinated proteins (Supporting Information Fig S1F) and proteasome activity was not increased (Supporting Information Fig S1C). In addition, increased levels of p62/SQSTM1 and a decreased ratio of LC3B-II/LC3B-I during hibernation indicated suppression of autophagy (Supporting Information Fig S1D and E).

Unexpectedly, phosphorylation of Akt at serine-478 was decreased (Fig 2A), yet downstream of Akt phosphorylation of p70^S6K^ and 4E-BP1, the main targets of mTOR signalling, were increased significantly during hibernation (Fig 2C). Phosphorylation of Akt at threonine 308 was not altered (Fig 2A and Supporting Information Fig S1B). The decrease in phosphorylated Akt (S478), in conjunction with Foxo3a inactivation and activation of mTOR signalling, demonstrates that Akt is not the sole mediator of protein synthesis or degradation in skeletal muscle during hibernation.

### SGK1 is a novel mediator of skeletal muscle homeostasis in hibernating mammals

**Figure 3:**
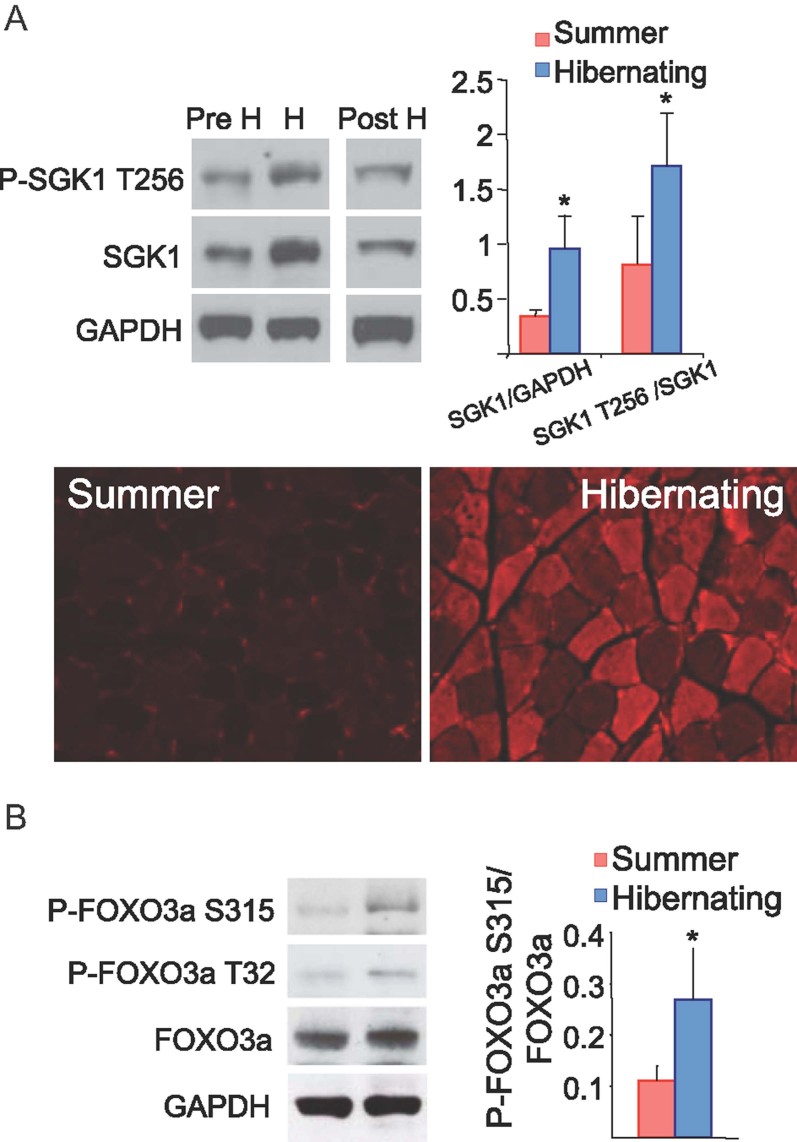
SGK1 in skeletal muscle is critical for preservation of skeletal muscle Western blot analyses and densitometry of quadriceps muscles from non-hibernating and hibernating squirrels demonstrates significant increases in total SGK1 and phosphorylated SGK1. Immunostaining of quadriceps muscle for total SGK1 shows upregulation during hibernation.Levels of phosphorylated Foxo3a in S315 and T32 are significantly increased during hibernation. Densitometric analysis of P-Foxo3a as a function of Foxo3a.

Serum- and glucocorticoid-inducible kinase 1 (SGK1) is a downstream target of the IGF-1/PDK-1 signalling cascade and shares a similar consensus phosphorylation site (RXRXXS/T) with Akt. Additionally, SGK1 phosphorylates Foxo3a at serine-253, serine-315 (S315) and T32 (Brunet et al, 2001) and is an alternative regulator of mTOR-mediated cell growth and survival in non-muscle cells (Aoyama et al, 2005). Therefore, we analysed SGK1 protein levels in the skeletal muscle of summer and hibernating squirrels. Hibernating muscles exhibited a significant increase in total and phosphorylated SGK1 that decreased to pre-hibernation levels once the animals emerged from torpor (Fig 3A). Immunostaining also showed increased SGK1 expression in hibernation (Fig 3A). As expected, increased phosphorylation of Foxo3a at S315 and T32 accompanied increase in levels of total and activated SGK1 (Fig 3B). Co-immunostaining revealed SGK1 localized mainly in fast, type II muscle fibers, which play a critical role in force generation during contractile activity (Supporting Information Fig S2A). In addition, cytoplasmic co-localization of SGK1 with phosphorylated Foxo3a (S253) was detected in type II muscle fibers of hibernating squirrels (Supporting Information Fig S2A) supporting our hypothesis that SGK1 modulates the inhibition of Foxo3a.

### SGK1 mediates skeletal muscle size and function in non-hibernating mammals

To analyse whether SGK1 plays a role in skeletal muscle of non-hibernating mammals, we performed expression analyses of SGK1 in various muscle types (Supporting Information Fig S2B). SGK1 is highly expressed in TA muscle, heart and diaphragm, with slightly lower levels of SGK1 protein expression in the mixed fiber type extremity muscles of quadriceps and gastrocnemius and even lower expression in soleus muscle.

In addition, we analysed SGK1 expression in transgenic mice that overexpress IGF-1 in skeletal muscle (*mIgf-1*). These mice develop skeletal muscle hypertrophy of primarily type II fibers (Musaro et al, 2001), accompanied by an increase in PDK1 and mTOR signalling without a parallel increase in phosphorylated Akt (Song et al, 2005) (Supporting Information Fig S2D). Our analyses indeed revealed increased SGK1 expression in type IIB hypertrophic muscle fibers (Supporting Information Fig S2C and E) and increased levels of activated phospho-SGK1 (Supporting Information Fig S2D), suggesting that SGK1 mediates muscle hypertrophy in *mIgf-1* mice.

**Figure 4:**
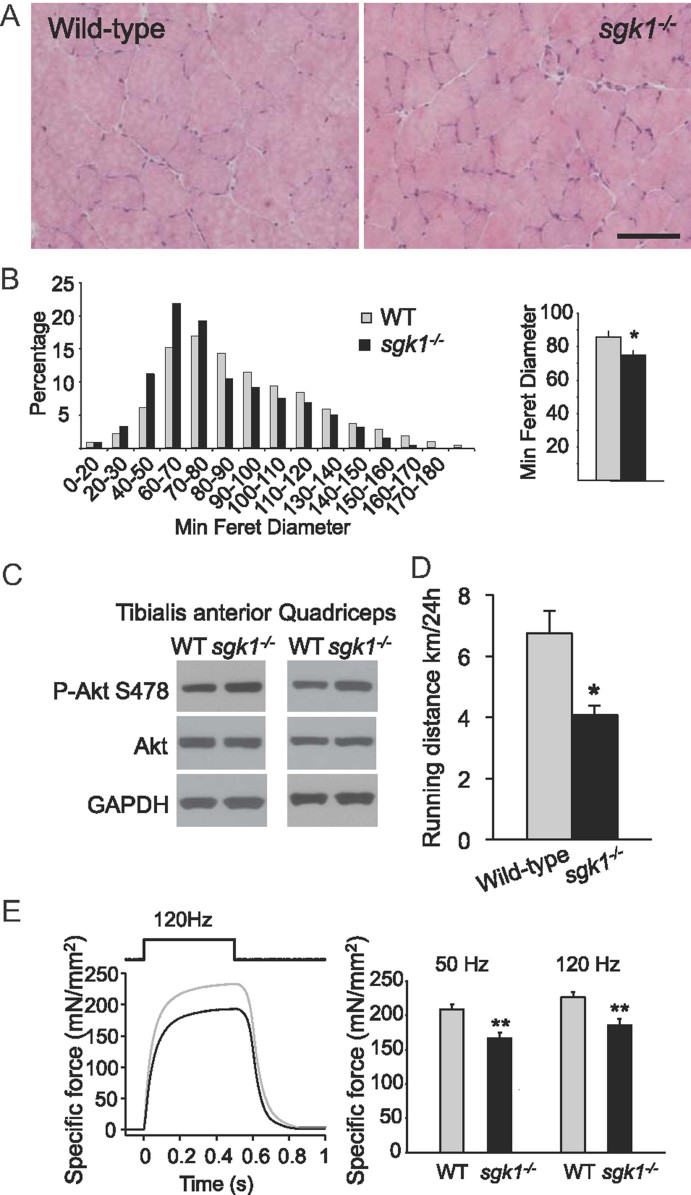
*Sgk1^−/−^* mice exhibit decreased muscle fiber size and impaired muscle function The morphology of tibialis anterior muscle as revealed by haematoxylin–eosin staining shows increased variation in muscle fiber size with numerous atrophic muscle fibers (scale bar 90 µm).Percentage distribution and mean minimum Feret's diameter in tibialis anterior muscle (*p* = 0.02).Increased levels of phosphorylated Akt (S478) in skeletal muscles of *sgk1*^*−*/*−*^ mice.*sgk1*^*−*/*−*^ mice exhibit decreased running distance after 36 days of exposure to a running wheel, (*p* = 0.01; *n* = 6 animals per group).Isometric force from soleus muscles of WT and *sgk1*^*−*/*−*^ mice during repetitive electrical stimulations for 500 ms. Amplitudes of isometric force from soleus muscles at two stimulation frequencies. Specific force is plotted in all cases. Mean values ± SD from *n* = 9 (*sgk1*^*−*/*−*^) and *n* = 10 (WT) independent animals (*p* = 0.01).

To further address the role of SGK1 in skeletal muscle maintenance, we evaluated skeletal muscle composition and function in mice lacking *sgk1*. These mice exhibit impaired renal sodium retention but the muscle phenotype has not been studied (Wulff et al, 2002). Mice deficient for *sgk1* showed no changes in body mass but revealed decreased weight of the TA muscles (Supporting Information Fig S2F). Histological evaluation of skeletal muscles of *sgk1*^*−*/*−*^ mice demonstrated increased variation of fiber size with numerous small and rounded myofibers (Fig 4A). Morphometric data confirmed that *sgk1*^*−*/*−*^ mice had decreased muscle fiber size in the TA, gastrocnemius and soleus muscles (Fig 4B and Supporting Information Fig S3E). Fiber type analysis of gastrocnemius and TA muscles did not reveal changes in fiber type composition (Supporting Information Fig S2G). Despite the decrease in muscle fiber size, skeletal muscle of *sgk1*^*−*/*−*^ mice showed an increase in phosphorylated Akt (Fig 4C and Supporting Information Fig S3A). Levels of phosphorylated Foxo3a at S315 were decreased in *sgk1*^*−*/*−*^ mice, whereas levels of phospho-Foxo3a at S253, LC3B and p62 were unchanged (Supporting Information Fig S3B).

In order to analyse the functional profile of these mice, *sgk1*^*−*/*−*^ mice completed 36 days of running wheel exercise and demonstrated a decrease in average and maximum speed (Supporting Information Table S1). This decrease was associated with a significant decline in running distance (Fig 4D).

*Sgk1*^*−*/*−*^ mice exhibited decreased specific isometric force in a physiological *in vitro* test suggesting that SGK1 plays an important role in skeletal muscle function (Fig 4E and Supporting Information Fig S3C and D).

We next tested the hypothesis that mice lacking *sgk1* are more susceptible to disuse and starvation atrophy. Thus, 2-month-old *sgk1*^*−*/*−*^ mice were exposed to 21 days of hindlimb immobilization and 48 h of starvation and compared to wild-type age matched littermates. Remarkably, *sgk1*^*−*/*−*^ mice demonstrated a significantly exaggerated response to immobilization and starvation (Supporting Information Fig S3F). In summary, our data reveal that SGK1 plays an important role in maintenance of muscle in steady-state and catabolic conditions such as disuse and starvation and indicate that increased levels of phosphorylated Akt are not able to fully compensate for the loss of SGK1.

Given these observations, we evaluated *akt1* knockout mice. Targeted deletion of *akt1* in mice has previously been shown to cause mild reduction in body size associated with slightly reduced muscle fiber size and strength (Goncalves et al, 2010). We detected increased SGK1 protein levels in *akt1*^*−*/*−*^ mice, which was associated with an increase in phosphorylation of Foxo3a at serine 315 (Supporting Information Fig S3G). Increased SGK1 signalling was not sufficient to compensate for the loss of *akt1*. Taken together, these results suggest that Akt and SGK1 both are necessary to ensure normal maintenance of skeletal muscle size and function.

### Overexpression of SGK1 protects against starvation and disuse muscle atrophy

**Figure 5:**
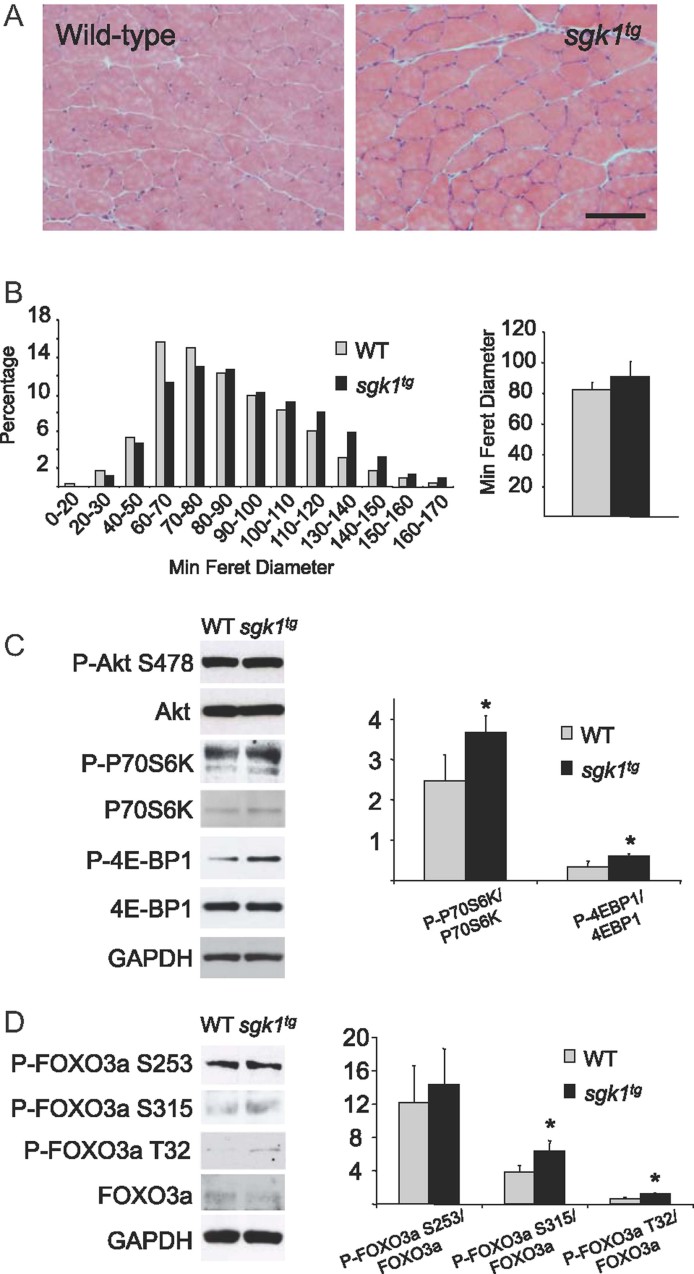
Characterization of skeletal muscle of SGK1 transgenic mice H&E staining of tibialis anterior sections from 2-month old control and transgenic mice shows no differences (scale bar 90 µm).Morphometric analyses of minimum Feret's diameter of TA muscle reveal no changes in fiber size distribution or mean fiber size.Western blots and densitometric analysis of muscle from transgenic and control mice. Downstream targets of the mTOR signalling cascade (p70S6K and 4EBP1) demonstrate significant upregulation of their phosphorylated forms in *sgk1*^*tg*^ mice.Western blot analyses and densitometry of phosphorylated FOXO3a and total FOXO3a of tibialis anterior muscles from control and transgenic mice (*n* = 4) show an increase of phosphorylation at S315 and T32.

Next, we analysed skeletal muscle of transgenic mice overexpressing the *sgk1* gene, which was modified by a point mutation (S422D) that renders the kinase constitutively active (Kobayashi & Cohen, 1999). RT-PCR analyses of the TA demonstrated an approximate 30% increase in expression of SGK1, which, in the context of the constitutively active kinase, provides an estimated threefold increase of SGK1 activation (Supporting Information Fig S4A and B). Histological and morphometric analyses of several skeletal muscles revealed no significant difference between the two groups (Fig 5A and B and Supporting Information Fig S4C). Interestingly, transgenic mice with inducible overexpression of Akt develop muscular abnormalities after Akt was induced and active during 6 months (Grumati et al, 2010), while constitutively active (ca)SGK1 transgenic adult mice did not show any pathological abnormalities at this age (Supporting Information Fig S4D). Fiber type immunostaining in gastrocnemius and TA muscles did not reveal changes in fiber type composition (Supporting Information Fig S4E). Biochemical analyses demonstrated increased abundance of phosphorylated p70^S6K^ and 4E-BP1 (Fig 5C). Furthermore, skeletal muscle of caSGK1 transgenic mice exhibited increased phosphorylation of Foxo3a at S315 and T32, similar to our findings in hibernating skeletal muscle (Fig 5D). Unexpectedly, analyses of autophagy markers in *sgk1* transgenic showed increased basal levels of LC3B-II and p62 (Supporting Information Fig S4F). Overall, our examination of these mice finds that overexpression of SGK1 does not cause any gross abnormal muscle phenotype.

**Figure 6:**
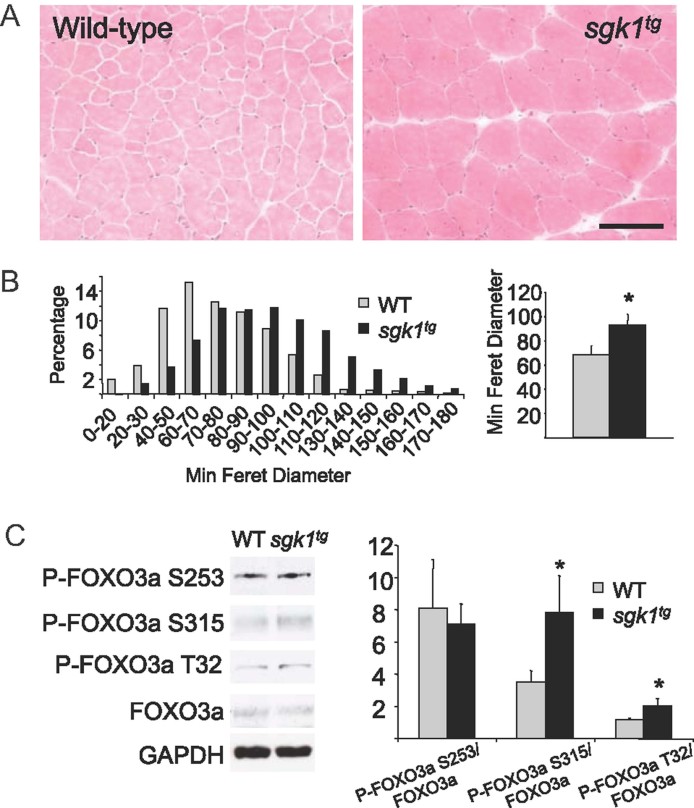
SGK1 protects against starvation-induced muscle atrophy H&E staining of tibialis anterior sections reveals no changes in muscle architecture (scale bar 90 µm).Morphometric analysis demonstrates a decrease in muscle fiber size of the wild-type control mice compared with *sgk1*^*tg*^ transgenic littermates (*p* = 0.0015).Western blot and densitometry analysis shows that levels of phosphorylated Foxo3a at S315 and T32 are increased in *sgk1*^*tg*^.

To test whether increased expression of SGK1 was sufficient to protect skeletal muscle against the catabolic stress of starvation, transgenic mice were fasted for 48 h. Remarkably, caSGK1 transgenic mice were protected against starvation-induced decrease in muscle fiber size when compared to age-matched littermate controls (Fig 6A and B). Previous biochemical analyses of fasted mice have shown decreased levels of phosphorylated Foxo3a, which promotes muscle atrophy via increased protein degradation and autophagy (Grumati et al, 2010; Mammucari et al, 2007). Our analyses demonstrated no measurable difference of phosphorylated Foxo3a at S253, but we found a significant increase of phosphorylated Foxo3a at S315 and T32 in starved mice overexpressing SGK1 (Fig 6C). We subsequently analysed gene expression levels of atrogin-1, MuRF-1 and Map1/LC3B by real-time PCR. Expression of atrogin-1 and MuRF-1 increased in wild-type and caSGK1 mice after starvation; however atrogin-1 levels in caSGK1 transgenic mice were significantly lower compared with wild-type. Wild-type mice had a significant increase in Map1/LC3B expression after fasting, which was not observed in caSGK1 transgenic mice (Supporting Information Fig S5A). Furthermore, starved caSGK1 transgenic mice showed a significantly smaller increase in the protein level of autophagy markers such as beclin-1 and LC3B-II when compared to fasted littermates control animals (Supporting Information Fig S5B). Given that autophagy has been implicated in loss of muscle mass during catabolic stress (Mammucari et al, 2007), our results indicate that inhibition of excessive autophagy contributes to the protection of starvation-induced loss of muscle mass in caSGK1 transgenic mice.

**Figure 7:**
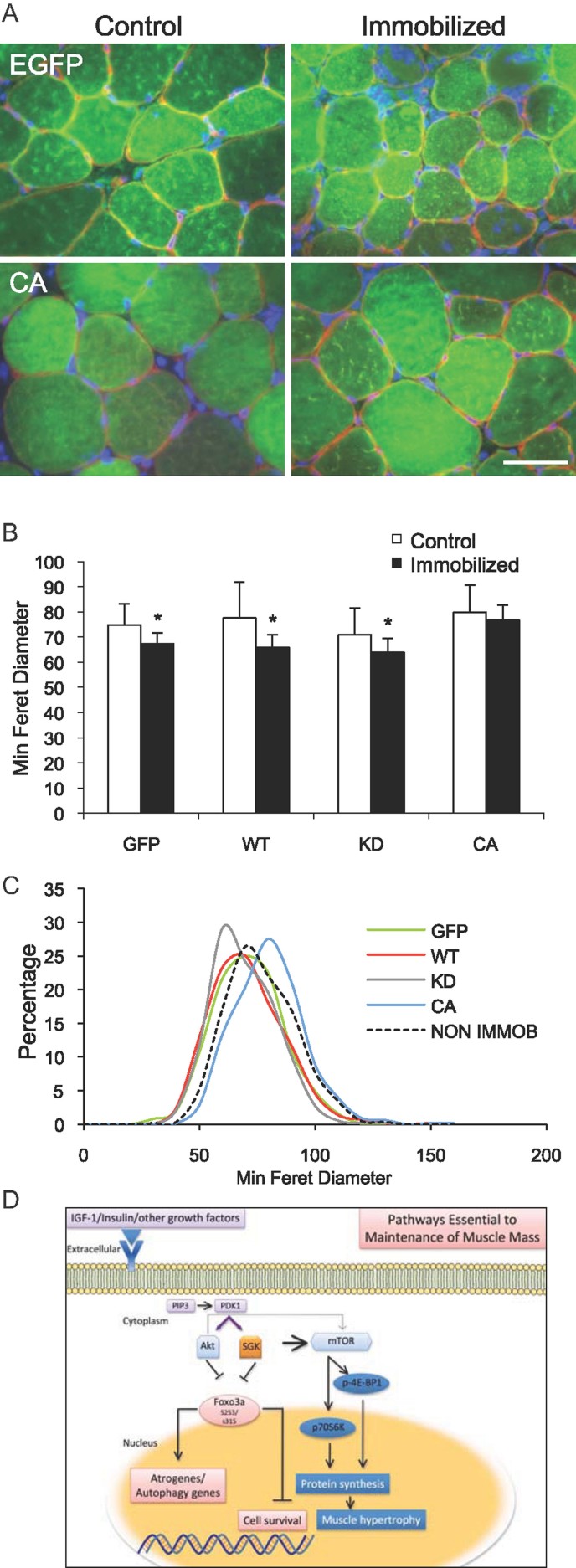
Electroporation of constitutively active *Sgk1* protects against immobilization atrophy Transfection of constitutively active *Sgk1* (CA) into immobilized tibialis anterior muscle (green) reveals increased fiber size diameter when compared to control, EGFP only transfected muscle fibers of immobilized tibialis anterior muscles (100 µm). Laminin γ-1 staining (red) outlines the basement membrane and blue staining marks nuclei (DAPI).Representation of the minimum Feret's diameter average ± SD (control and immobilized; *n* = 9–10 per group; 800–950 fibers were measured) (*p = 0.029, *p = 0.034, *p = 0.047, *p* = 0.43, respectively).Percentage distribution of the minimum Feret's diameter of tibialis anterior muscle immobilized and transfected with eGFP (GFP), wild-type *Sgk1* (WT), kinase dead *Sgk1* (KD) and constitutively active *Sgk1* (CA) compared to non-immobilized control TA muscle (black dotted line).Schematic representation of pathways involved in the preservation of skeletal muscle mass during prolonged periods of immobilization. SGK1, in addition to Akt, mediates protein degradation via phosphorylation of Foxo3a, with the subsequent inhibition of proteolysis and autophagy and protein synthesis through the activation of mTOR.

In order to assess whether SGK1 expression protects against targeted disuse atrophy, we transfected TA muscles via electroporation with plasmids encoding *Sgk1* WT (*Sgk1*^*WT*^), Kinase Dead (*Sgk1*^*KD*^) and Constitutively Active *Sgk1* (*Sgk1*^*CA*^) all fused to EGFP. One hindlimb was immobilized by stapling (Burks et al, 2011), and the plasmids were injected into both the immobilized and the contralateral non-immobilized muscles. After 9 days, both TA muscles were assessed for plasmid expression and morphometric measurements. Co-immunostaining for laminin γ-1 ensured that plasmid expression was confined to skeletal muscle fibers and not within extracellular space. Gene transfection efficiency was determined by calculating the cross sectional area of GFP positive muscle fibers expressed as a percentage of the total cross sectional area (Schertzer et al, 2006). All constructs revealed a transfection efficiency of 58.6–78.5% (Supporting Information Fig S6D). Transfection of contralateral, non-immobilized TA muscles did not show any significant changes in muscle fiber size or morphology with any of the plasmids injected (Supporting Information Fig S6B). However, in the immobilized limb transfection of *Sgk1*^*CA*^ completely prevented muscle atrophy when compared to GFP only, *Sgk1*^*WT*^ and *Sgk1*^*KD*^ (Fig 7A–C and Supporting Information Fig S6A). Importantly, skeletal muscle atrophy was only prevented by transfection of the constitutively active form of *Sgk1*.

Analysis of the expression of Foxo3a and members of the mTOR pathway (Supporting Information Fig S6C) demonstrated an increased phosphorylation of Foxo3a in S253 and S315 and p-4EBP1 in muscles overexpressing SGK1^CA^. These results further support our observation that immobilized muscles overexpressing SGK1^CA^ are protected against muscle atrophy.

This experiment suggests that SGK1 requires activation to exert its beneficial impact on the regulation of skeletal muscle mass. Together, our data shows that overexpression of SGK1 ameliorates atrophy in immobilization and starvation. These findings further support our hypothesis that SGK1 is an important mediator of skeletal muscle homeostasis.

## DISCUSSION

Maintaining a homeostatic balance between protein synthesis and degradation protects against loss of skeletal muscle mass, plasticity and function. We demonstrate that increased expression of SGK1, leading to inhibition of Foxo3a activity and accompanied by increased expression of the mTOR signalling cascade, preserves skeletal muscle mass during prolonged immobilization and starvation during hibernation. Furthermore, functional and molecular analysis of SGK1 in several non-hibernating animal models identifies SGK1 as a novel mediator of skeletal muscle homeostasis and function.

Current paradigm emphasizes the role of Akt in the IGF-1/PI3K pathway and its downstream targets, Foxo3a and mTOR, thereby regulating skeletal muscle cell size and function (Glass, 2003). Analysis of the naturally occurring extreme physiological state of hibernation shows that phosphorylation of Foxo3a at serine-253, -315 and T32 is associated with increased phosphorylation of mTOR targets, p70^S6K^ and 4E-BP1, independent of Akt activation. Instead hibernating squirrels demonstrate increased expression and activation of SGK1. SGK1 returns to baseline levels after hibernating squirrels emerge from hibernation, suggesting that this increase is necessary to protect skeletal muscle against atrophy during hibernation. Biochemical *in vitro* analyses suggest that SGK1 phosphorylates Foxo3a at serine-315 with high affinity, serine-253 with less affinity than Akt, and T32 with equal affinity as Akt (Brunet et al, 2001). Furthermore, SGK1 has been shown to regulate mTOR signalling, cell survival and hypertrophy in cardiomyocytes *in vitro* (Aoyama et al, 2005). In accordance with these *in vitro* studies, our *in vivo* analyses indicate that, in the absence of increased activation of Akt, SGK1 phosphorylates Foxo3a at serine-253, serine-315 and T32 and that it mediates activation of the mTOR signalling cascade and its downstream targets in skeletal muscle (Fig 7D).

Beyond its protective role in hibernating animals, SGK1 plays a critical role in muscle homeostasis and function. Analyses of mice lacking *sgk1* reveal decreased muscle mass and fiber size with an otherwise normal structural appearance. *Sgk1*^*−*/*−*^ mice show reduced phosphorylation levels of Foxo3a at serine-315 but normal levels of Foxo3a at serine-253. These findings are likely due to a compensatory increase in phosphorylated Akt levels in *sgk1*^*−*/*−*^ mice. Despite this increase, *sgk-1*^*−*/*−*^ mice have impaired muscle function as shown by diminished performance on a running wheel as well as decreased specific muscle force generation *in vitro*. Furthermore, *sgk-1*^*−*/*−*^ mice demonstrated a remarkably exaggerated response to disuse and starvation challenge. Previous research shows that increased activation of Akt improve muscle strength and metabolism of fast muscle fibers (Izumiya et al, 2008). The compensatory increase of Akt in *sgk-1*^*−*/*−*^ mice fails to restore normal function and protect against disuse and starvation atrophy indicating that SGK1 may act via additional, Foxo3a/mTOR independent, mechanisms to mediate skeletal muscle homeostasis. These results prompted us to analyse expression of SGK1 and its downstream targets in mice deficient for *akt1*. These mice, similar to *sgk1*^*−*/*−*^ mice, have mildly decreased muscle fiber size with decreased muscle strength (Goncalves et al, 2010). Our analyses revealed increased expression of SGK1 together with increased phosphorylation of Foxo3a at serine-315. Skeletal muscle abnormalities of *sgk1*^*−*/*−*^ and *akt1*^*−*/*−*^ mice suggest that neither Akt nor SGK1 can completely compensate for the loss of the other, demonstrating that Akt and SGK1 are necessary to ensure normal skeletal muscle size and function. Indeed, preliminary experiments in our laboratory show that knockdown of SGK1 expression in wild-type adult mice postnatally, induces a compensatory increase of Akt without a noticeable short-term impact on skeletal muscle morphology and function. We are currently in the process of evaluating the long-term impact of knockdown of SGK1 in adult, postnatal skeletal muscle.

Since SGK1 seems to play a protective role in hibernating squirrels and has a compensatory hypertrophic response in non-hibernating mammals, we analysed transgenic mice with constitutively active *sgk1* expression (Arteaga et al, 2007). Biochemical analyses of these mice demonstrate increased phosphorylation of Foxo3a at serine-315 as well as increased levels of additional downstream targets of SGK1. The expression profile of muscle of mice overexpressing SGK1 was remarkably similar to our findings from muscle of hibernating squirrels. Morphometric analysis of skeletal muscles from SGK1 transgenic mice did not show a significant increase in skeletal muscle fiber size; in contrast, adult transgenic mice overexpressing Akt1 during the first 6 months of life develop significant hypertrophy of type II muscle fibers (Izumiya et al, 2008; Lai et al, 2004). These findings might be related to the lower-fold increase of SGK1 and/or the increase on autophagy in *sgk1* transgenic mice as compared to the expression profile of Akt in transgenic Akt mice. Importantly for therapeutic potential, transgenic Akt mice develop dystrophic abnormalities of skeletal muscle after 6 months (Grumati et al, 2010), which is not seen in our *sgk1* transgenic mice, suggesting that the milder increase of SGK1 expression may not interfere with normal skeletal muscle homeostasis even over a prolonged period of time.

*Sgk1* transgenic mice demonstrate protection against loss of muscle mass under the systemic catabolic stress situation of prolonged starvation, which causes significant muscle atrophy (Boehm et al, 2007). This phenotypic benefit was associated with an expected increase in phosphorylation of Foxo3a at serine-315 and T32. In contrast to control animals, caSGK1 transgenic mice show a lack of increase in gene and/or protein expression of autophagy mediators. These findings are of particular significance as autophagy has been reported to contribute to loss of muscle mass during starvation (Mammucari et al, 2007; Zhao et al, 2007). Our results indicate that inhibition of excessive autophagy protects against skeletal muscle atrophy during catabolic stress and suggest that SGK1 may regulate autophagy. However, given that transgenic mice exhibit ubiquitous expression of *sgk1*, we cannot exclude that other organ systems may have an impact on the protection against skeletal muscle atrophy.

Electroporation of TA muscle allowed us to assess the function of increased SGK1 expression in postnatal adult muscle fibers eliminating developmental, metabolic or systemic effects. Our experiments demonstrate that increased expression of constitutively active *Sgk1*, but not wild-type *Sgk1*, completely protected TA muscles from immobilization-induced atrophy. Although a number of stimuli such as growth factors and hormones are known to cause genomic upregulation of SGK1 (Lang et al, 2006), activation of SGK1 is necessary to exert its biological function (Kobayashi & Cohen, 1999).

Therefore, our results indicate that SGK1 in conjunction with Akt mediates muscle homeostasis and that activation of SGK1 is necessary to preserve skeletal muscle mass under stressful conditions. Our findings emphasize the potential of SGK1 as a novel therapeutic target to combat loss of skeletal muscle mass and function in neuromuscular conditions involving skeletal muscle atrophy and hypertrophy. Finally, the overarching conceptual advance that this work represents is the promise that molecular analyses of naturally occurring extreme physiological states, such as hibernation, can reveal unanticipated pathogenic mechanisms and rational treatment strategies.

## MATERIALS AND METHODS

### 13-lined ground squirrels (*I. tridecemlineatus*)

All experimental procedures with 13-lined ground squirrels were approved by the Animal Care and Use Committee of Johns Hopkins University School of Medicine. All animal procedures conformed to federal welfare guidelines and were pre-approved by the relevant IACUC. Hibernation-naïve euthermic weanlings of both sexes were obtained in July from the captive breeding colony at the University of Wisconsin Oshkosh (Vaughan et al, 2006) and supplied with food and water *ad libitum*. Squirrels were injected with ketamine before dissection.

When first torpor was observed, food and water were removed and the squirrels were transferred to a dark hibernaculum maintained at ∼4°C. Torpor was monitored daily by animal cage inspection in the dark and displacement of wood chips was used to determine arousal during hibernation. For our experiments, squirrels in the torpid, hypothermic state were sacrificed 4–5 months after first immergence into torpor (*n* = 10). We also assessed skeletal muscle size of animals after 2–3 months of torpor (*n* = 6) and did not see any signs of muscle atrophy (data not shown). In April, when the squirrels emerged naturally from winter hibernation, they were returned to a warm room and food and water were reinstated. The muscles collected from each animal included the diaphragm, quadriceps, gastrocnemius, TA and biceps. Muscle samples were rapidly processed by flash freezing, or homogenization for a variety of analyses.

### Skeletal muscle morphometry

Skeletal muscle was obtained and flash frozen in OCT and cold isopentane. Samples were cut at 10 µm per section. Skeletal muscle morphometry was performed following standard methods (Briguet et al, 2004; Cohn et al, 2007) with some modifications. We determined fiber diameter and the minimal Feret's diameter in quadriceps muscle using Nikon's NIS elements BR3.0 software (Laboratory Imaging, Nikon). A minimum of 2000 fibers per animal was analysed. All images were taken using an Eclipse 80i microscope (Nikon, Inc.).

### Histology and immunofluorescence

Skeletal muscle was flash frozen in cooled isopentane and mounted in OCT compound (Tissue-Tek, Sakura Finetek). Subsequently, we stained 10 µm sections with hematoxylin and eosin following standard protocols (Cohn et al, 2007). Indirect immunofluorescence was performed on frozen sections blocked with 10% normal goat serum or 5% bovine serum albumin for 1 h (Cohn et al, 2007). Primary antibodies used for immunofluorescence included polyclonal anti-dystrophin c-terminus (Santa Cruz), monoclonal rat anti-laminin γ-1 polyclonal anti-SGK and anti-phospho-FOXO3a S253, LC3B and LC3B XP (Cell Signalling). Fiber-typing antibodies were kindly provided by Dr. Hourdé (Agbulut et al, 2009). For fiber-type analyses, sections were air-dried, washed in phosphate-buffered saline (PBS) with 0.1% (v/v) Tween-20 (PBS-T) and stained for laminin γ-1 or for the different MyHC isoforms, with antibodies harvested from hybridoma cell lines obtained from the American Type Culture Collection (Manassas, VA, USA): BA-D5 (IgG2b, anti-MHCI), SC-71 (IgG1, anti-MHCIIa), BF-F3 (IgM, anti-MHCIIb). The sections were incubated at room temperature for 1 h in a blocking solution (bovine serum albumin (BSA) 1%, sheep serum 1%, triton X-100 0.1%, sodium azide 0.001%). Sections were then incubated at room temperature for 2 h with anti-MyHC-I (BA-D5, 2:3) and anti-MyHC-IIA (SC-71, 1:3). Sections were then incubated overnight at 4°C with anti-MyHC-IIb (BF-F3, 1:1). Sections were washed as before and secondary antibodies were applied for 1 h at a dilution of 1:400. Alexa 488 anti-mouse IgG2b, Alexa 350 anti-mouse IgG1 and Alexa 594 anti-mouse IgM were obtained from Invitrogen. Nuclear staining was performed with DAPI and the glass slides were mounted with Vectashield Mounting Medium (Vector Laboratories).

### Immunoblot analysis

To determine protein expression, flash-frozen quadriceps muscles were lysed with ice-cold lysis buffer (Nonidet P-40 1%, glycerol 10%, NaCl 137 mM, Tris–HCl pH 7.5 20 mM) containing protease (Complete Mini, EDTA-free, Roche) and phosphatase inhibitors (PhosSTOP, Roche). Tissue lysate (20 µg) was electrophoresed in Bis-Tris or Tris-glycine gels and transferred onto nitrocellulose membranes via standard procedures. Membranes were incubated with primary antibody diluted in blocking solution overnight at 4°C. Primary antibodies used were: anti-Akt, anti-phospho-Akt S473, anti-phospho-Akt T308, anti-phospho-GSK3β, anti-phospho-SGK T256, anti-4EBP1, anti-phospho-4EBP1 S65, anti-p70S6K, anti-phospho-p70S6K T389, anti-FOXO3a, anti-phospho-FOXO3a S253 and T32, anti-Beclin, anti-ATG7, LC3B XP and anti-LC3B (Cell Signalling); anti-GAPDH, anti-phospho-SGK T256 (Santa Cruz); anti-phospho-SGK S422 (Santa Cruz and Abcam); anti-p62/SQSTM1 (Abcam and Cell Signalling); anti-SGK (Cell Signalling and Upstate); anti-phospho-FOXO3a S253 (Millipore); anti-GSK3β (Upstate), anti-ubiquitin (DakoCytomation). The antibody anti-phospho-FOXO3a S315 was kindly provided by Dr. Anne Brunet (Stanford University). After washing, immunoreactive bands were detected with HRP-conjugated secondary antibodies (Amersham) and visualized with Supersignal West Dura or Supersignal West Femto Substrates (Pierce). Densitometry analysis was performed using ImageJ 1.37 (National Institutes of Health, USA). Statistical analysis of Western blot results is shown in Supporting Information Table S2.

### Proteasome activity

The proteasome activity was measured using fluorogenic peptide substrates LLVY (succinyl-leu-leu-val-AMC) following the manufacturer's protocol (Chemicon). Quadriceps muscles were homogenized in lysis buffer (Tris–HCl pH 7.2 50 mM, EDTA 1 mM, KCl 100 mM, MgCl_2_ 5 mM, ATP 1.8 mM), and centrifuged at 700*g* for 10 min. The supernatant was centrifuged for 10 min at 15,000*g* and glycerol was added to a final concentration of 10% (Klaude et al, 2007). The proteasome was isolated and its activity was measured immediately; aliquots were stored at −80°C to repeat this measurement the next day. Triplicates of each sample were assayed over two consecutive days. Fluorescence was measured in a SpectraMax GeminiXS (Molecular Devices, Sunnyvale, California) with an excitation wavelength of 380 nm and an emission wavelength of 460 nm.

### Real-time PCR

Total RNA was isolated with TRIzol (Invitrogen) and treated with a Turbo DNA free Kit (Ambion). RNA (1.5 µg) was reverse transcribed using TaqMan RT reaction (Applied Biosystems). PCR reactions were performed using SYBR Green PCR Master Mix (Applied Biosystems) and the 7900HT Sequence System ABI PRISM (Applied Biosystems) Real Time PCR system. Genes were quantified using GAPDH. All the sequences of the primers are mouse sequences. The primers that were used are: atrogin-1 forward: -5′gcaaacactgccacattctctc3′, reverse: 5′cttgaggggaaagtgagacg3′; beclin forward: 5′ggccaataagatgggtctga3′, reverse: 5′cactgcctccagtgtcttca3′; GAPDH forward: 5′caccatcttccaggagcgag3′, reverse: 5′ccttctccatggtggtgaagac3′; MAP1-LC3b forward: 5′cactgctctgtcttgtgtaggttg3′, reverse: 5′tcgttgtgcctttattagtgcatc3′; murf-1 forward: 5′ggtgcctacttgctccttgt3′, reverse: 5′ctggtggctattctccttgg3′.

### Running wheel protocol

To explore whether SGK1 influences physical performance, *sgk1*^*−*/*−*^ mice (*n* = 6) and *sgk1*^*+*/*+*^ mice (*n* = 6) were placed into individual cages which contained a running wheel (diameter: 12 cm) consisting of stainless steel and equipped with an electronic speedometer (Sigma Sport 600, Sigma Sport, Neustadt, Germany). The running wheels were totally wrapped by a plastic ribbon. The speedometer automatically determined the running distance (km/24 h), the time period during which the running wheel was used allowing the calculation of average and maximal speed (km/h). Average values were calculated from the performance during at least 14 days.

### Muscle force measurements

Contractile properties were measured on soleus muscles from 90 to 100 days old mice. All experiments were performed with male wild-type and SGK1^*−*/*−*^ mice of the 129/J strain (Wulff et al, 2002). Tendons were fixed with surgical twines (Prolene 6 × 0) and the muscles mounted in a recording chamber. The proximal tendon was connected to the transducers hook (tppe KG4, Scientific Instruments, Heidelberg, Germany). The chamber was continuously perfused with Krebs-Henseleit solution (at pH 7.4) containing (in mM): 137 NaCl, 24 NaHCO_3_, 5 KCl, 2 CaCl_2_, 1 NaH_2_PO_4_, 1 MgSO_4_ and 11 d-glucose. The solution was continuously was bubbled with 95% O_2_ and 5% CO_2_. The pH was continuously controlled with a minitrode (pH 0–14, Hamilton Bonaduz AG, Bonaduz, Switzerland). All experiments were performed at 25°C. Muscles were electrically stimulated trough platinum electrodes that were placed in the recording chamber. Single or repetitive supramaximal stimuli of 50 V and 1-ms duration were applied (STIM8 stimulator, Scientific Instruments). Isometric forces were registered with a digital 2-channel storage oscilloscope (Tektronix DPO 2012, Tektronix Inc., Beaverton, OR, USA). The collected data were read out and stored on a computer.

Before experiments were started, the optimal muscle length was ascertained. For that purpose single twitches were elicited every 30 s by 1-ms pulses and the length of the soleus muscle was step by step increased at intervals of 250 µm. The force–length relation followed roughly a parable from which the optimal muscle length was calculated. After an hour of rest the length optimization was repeated and the muscle length adjusted to the length of the maximal force. Then, responses to single stimuli and to repetitive 500-ms lasting series of pulses at 10, 50 and 120 Hz were recorded. The recordings were followed by a fatigue protocol consisting of 500-ms lasting tetani (stimulation frequency 50 Hz) repeated every 2 s for 340 s. Single twitches were analysed for time to peak, half relaxation time and peak force. Tetani were analysed for maximum tetanic force. All data on muscle force are presented as specific force, that is force per cross sectional area of the muscles.

### *mIgf-1* and SGK1 transgenic mice

Transgenic mice overexpressing IGF-1 in skeletal muscle were kindly provided by Dr. Nadia Rosenthal. Generation and extensive characterization of skeletal muscle of *mIgf-1* transgenic mice has been previously published (Musaro et al, 2001). A BAC containing 180 kbp of mouse genomic sequence that includes the full SGK1 gene but no other known or predicted genes was obtained from the BACPAC Resources Center (Children's Hospital Oakland Research Institute, Oakland, CA). The BAC was modified using homologous recombination in *Escherichia coli* (Lalioti & Heath, 2001) to add three copies of the HA epitope in the c-terminus of SGK1 and a point mutation (S422D) that renders the kinase constitutively active (Kobayashi & Cohen, 1999). The integrity of the modified BAC was checked by DNA sequencing of the insert ends and by Southern blotting. The genomic insert was excised by digestion with *Not*I, purified by field inversion gel electrophoresis followed by digestion with β-agarase and used for pronuclear injection of (C57BL/6J X SJL/J) F2 embryos. Genotyping was performed by PCR analysis of genomic DNA using primers 5′-GGAAGCAGCAGAAGCCTTCCTCGG-3′ and 5′-GACTGCCAAGCTTCCAGGTGTGC-3′, which flank the stop codon and produce a 186 bp product with wild-type SGK1 and a 267 bp product with Tg.SGK1 due to the insertion of three consecutive copies of the HA-epitope. Founder animals harbouring the transgene were crossed with C57BL/6 mice. Mice homozygous for the transgene were obtained by crossing heterozygous animals.

### Starvation experiment

Mouse chow was removed from the cage in the morning, mice were maintained for 48 h with no food but free access to water.

### Electroporation

SGK1 was amplified and fully sequenced from Addgene plasmid 20628 (Boehm et al, 2007). The first 60 aminoacids were deleted and replace with a start codon (ATG) to decrease the localization and degradation by the endoplasmic reticulum (Arteaga et al, 2006). The Kinase Dead *Sgk1* (*Sgk1*^*KD*^) with a point mutation (K127A) and Constitutively Active *Sgk1* (*Sgk1*^*CA*^) with a point mutation (S422D) were made with QuickChange II XL Site-Directed Mutagenesis Kit (Stratagene). The PCR fragments were cloned in pEGFP-C1 vector (Clontech). Plasmid expression was tested in HEK293 and C2C12 cells using antibodies rabbit anti-SGK1 (Cell Signalling) and mouse anti-GFP (Abcam).

Skeletal muscle electroporation was carried out using BTX 830 square wave electroporator (BTX-Harvard Apparatus, Holliston, MA, USA) using platinum coated paddles. After removal of the animal's hindlimb hair, 40 µl of bovine hyaluronic acid (0.3 U/µl in sterile and endotoxin-free 0.9% NaCl solution; Sigma–Aldrich, St. Louis, MO, USA) was injected in 10 µl aliquots up the length of the TA muscle using a 26 gauge needle and syringe. Hyaluronic acid pretreatment prior to electroporation has been shown to increase transfection efficiency. Two hours later, animals were sedated with ketamine and xyalizine. The TA from both hindlimbs was injected with 40 µl of plasmid encoding for *Sgk1* WT (*Sgk1*^*WT*^), Kinase Dead (*Sgk1*^*KD*^), Constitutively Active *Sgk1* (*Sgk1*^*CA*^) fused to eGFP (1 µg/µl plamid in 0.9% NaCl solution) in 10 µl aliquots up the length of the muscle (*n* = 10 per group). Control animals were injected with pEGFP or hyaluronidase (*n* = 10 per group). Immediately following plasmid injection, the muscle was electroporated with five 25 ms pulses of 150 V of electric current.

**The paper explained**

PROBLEM:

Skeletal muscle atrophy (in particular during aging) has severe consequences on the quality of life of the human population and also places a significant financial burden on health care systems. Currently, there are no treatments available to protect loss of muscle mass in a variety of inherited and acquired forms of myopathy. Loss of muscle mass results from perturbed homeostasis of protein synthesis and degradation. To explore the critical contribution of molecular pathways involved in the preservation of muscle mass, we analysed 13-lined ground squirrels, a naturally occurring, hibernating animal that is protected from muscle atrophy despite prolonged periods of immobilization and starvation.

RESULTS:

Previous evidence suggests that Akt is the only determinant and regulator of Foxo3a-mediated atrophy/autophagy, mTOR signalling and cell survival in skeletal muscle. Using hibernating muscle as a tool to understand skeletal muscle homeostasis, we have tested and ultimately refuted this longstanding tenant of muscle biology. We demonstrate that Foxo3a-mediated inhibition of muscle atrophy/autophagy, activation of the mTOR signalling cascade and muscle cell survival are regulated independently of Akt activation. Instead, analysis of the hibernating squirrel muscles has allowed us to identify serum/glucocorticoid-induced kinase 1 (SGK1) as a novel and central factor, mediating skeletal muscle preservation during hibernation. Insights derived from our hibernating model can be generalized, as we were able to establish that SGK1 plays a critical role in muscle function beyond hibernation. We demonstrate that mice lacking *sgk1* exhibit skeletal muscle atrophy associated with decreased muscle strength *in vivo* and *in vitro*. Furthermore, we show that overexpression of constitutively active SGK1 protects against disuse atrophy and starvation-induced muscle atrophy.

IMPACT:

Our results have identified a novel therapeutic target to combat loss of skeletal muscle mass in a variety of conditions associated with muscle atrophy and degeneration. The overarching conceptual advance that this work represents is the promise that molecular analyses of naturally occurring extreme physiological states, such as hibernation, can reveal unanticipated pathogenic mechanisms and rational treatment strategies.

Right leg was immobilized after electroporation by stapling the hindlimb (Burks et al, 2011). After 10 days, TA was harvested, flash frozen and sectioning. Immunostaining was performed using rat anti-laminin γ-1 antibody (Cell signalling). Double stained fiber area and the minimal Feret's diameter analysis was performed using ImageJ 1.37 (National Institutes of Health, USA).

### Statistical analysis

All values are expressed as mean ± SD. Significance was determined by unpaired Student's *t*-tests or by one or two-way ANOVA followed by the Student–Newman–Keuls test. Significance was set at *p* ≤ 0.05.

## Supplementary material

Detailed facts of importance to specialist readers are published as ”Supporting Information”. Such documents are peer-reviewed, but not copy-edited or typeset. They are made available as submitted by the authors.
