# Supplementary material for: Activation of serum/glucocorticoid-induced kinase 1 (SGK1) is important to maintain skeletal muscle homeostasis and prevent atrophy
Source: EMBO Mol Med. 2012 Nov 19;5(1):80–91. doi: 10.1002/emmm.201201443 (PMC3569655; doi:10.1002/emmm.201201443)
Supplement: Supplementary file 1 [file emmm0005-0080-SD1.pdf]

EMBO Molecular Medicine

Manuscript EMM-2012-01443

Activation of Serum/Glucocorticoid-Induced Kinase 1 (SGK1) is important to maintain skeletal muscle homeostasis and prevent atrophy

Eva Andres-Mateos, Heinrich Brinkmeier, Tyesha N. Burks, Rebeca Mejias, Daniel C. Files, Martin Steinberger, Arshia Soleimani, Ruth Marx, Jessica L. Simmers, Benjamin Lin, Erika Finanger Hedderick, Tom G. Marr, Brian M. Lin, Christophe Hourdé, Leslie A. Leinwand, Dietmar Kuhl, Michael Föller, Silke Vogelsang, Ivan Hernandez-Diaz, Dana K. Vaughan, Diego Alvarez de la Rosa, Florian Lang and Ronald D. Cohn

Corresponding author: Ronald D. Cohn, Johns Hopkins University School of Medicine

Review timeline:

Submission date:	05 April 2012
Editorial Decision:	21 May 2012
Revision received:	14 August 2012
Editorial Decision:	10 September 2012
Revision received:	25 September 2012
Accepted:	28 September 2012

Transaction Report:

(Note: With the exception of the correction of typographical or spelling errors that could be a source of ambiguity, letters and reports are not edited. The original formatting of letters and referee reports may not be reflected in this compilation.)

1st Editorial Decision 21 May 2012

Thank you for the submission of your manuscript "Activation of Serum/Glucocorticoid-Induced Kinase (SGK) is sufficient to maintain skeletal muscle homeostasis and prevent atrophy" to EMBO Molecular Medicine and please accept my apologies for the delayed reply. We have now finally heard back from the three referees whom we asked to evaluate your manuscript. You will see that they find the topic of your manuscript potentially interesting. However, they also raise significant concerns about the study, which should be addressed in a major revision of the manuscript.

You will see that reviewer #3 is positive about the study, while reviewers #1 and #2 are more reserved. In particular, reviewer #2 feels that muscle-specific gain- and loss-of-function of Sgk1 should be further investigated and notes that well established models of atrophy and hypertrophy should be used. In addition, reviewer #1 highlights that the investigation of the proteolytic system should be strengthened.

Given the balance of these evaluations, we feel that we can consider a revision of your manuscript if you can convincingly address the issues that have been raised within the time constraints outlined below.

Revised manuscripts should be submitted within three months of a request for revision. They will

otherwise be treated as new submissions, unless arranged otherwise with the editor.

I look forward to seeing a revised form of your manuscript as soon as possible.

Yours sincerely,

Editor
EMBO Molecular Medicine

***** Reviewer's comments *****

Referee #1:

The group of Cohn RD and coauthors is presenting an interest study about the mechanism that protects ground squirrel from atrophy during hibernation. The authors found that Serum and Glucocorticoid inducible Kinase (SGK) is activated during hibernation while Akt is not. The authors validate the involvement of SGK by using SGK knockout and SGK transgenic mice. SGK knockout mice show a decrease in myofiber size while the transgenic mice are protected from muscle atrophy. Activation of SGK inhibits the activation of the autophagy-lysosome and ubiquitin-proteasome proteolytic systems. The paper is of absolute interest for biologists, medical doctor and physiologists. Moreover, having found that SGK is an nticatabolic factor open the field to the development of new drugs to counteract muscle wasting and to further investigations on the mechanisms of these actions. However, the paper needs further experiments to clarify the mechanisms of SGK action and some controls. The following points should be considered by the authors.

Point 1: The authors should better characterize the squirrel muscles. Is the proportion of beta-oxidative versus glycolitic fibers changed during hibernation? Are the level of PGC1a-PGC1b also affected by hibernation? This is an important aspect since b-oxidative fibers are known to be resistant to muscle atrophy.

Point 2: Figure 2, the characterization of the proteolytic system needs additional data. Is the total amount of ubiquitinated proteins changed in squirrel during hibernation? Despite the expression of the autophagy genes, authors should determine the level of LC3 lipidation (LC3II/LC3I ratio)and p62/SQSTM1 (protein/mRNA). Moreover, the amount of autophagosome revealed as LC3 positive puncta should be also determined. In the case that LC3 and p62 antibodies are not available for squirrel the authors might use electron microscopy to determine whether autophagy is present during hibernation. Moreover these analyses should be applied to muscles of SGK1 transgenic and knockout mice.

Point 3: The authors should quantify the phosphorylation status of Akt at threonin 308. This is important since recent findings underline a potential different role of the phopshorylation sites on Akt activity on downstream targets.

Point 4: Figure 3C is unnecessary for the story and should be removed. Indeed the finding that total SGK is increased in dystrophin-deficient myofiber does not mean that SGK has a functional relevance. If the authors want to show these data they have to prove that SGK is more phosphorylated in hypertrophic fibers and cross the SGK transgenic with mdx mice and show amelioration. Personally, I do not think that the dystrophin deficient data fit with the story. Indeed the mechanisms that prevent muscle wasting during atrophy are different from the mechanisms that protect from degeneration. An additional minor point is related to the bibliography. The first paper that describes a beneficial effect of Akt on dystrophin-deficient muscles is Blaauw et al. 2008, Hum Mol Genet. and not Peter et al. 2009.

Point 5: Figure 4A, the picture is of poor quality and it is difficult to determine the changes in morphology (small rounded. Are CSA of gastrocnemius and soleus muscles affected by the absence

of GSK? The authors focused their attention on exercise alterations and force measurements that are indeed important aspects. However it would be of interest to know whether these mice are more prone to atrophy in catabolic conditions. In fact the classical knockout approach induce an early and systemic deletion of the studied gene. Therefore the decrease of CSA might be a consequence of a defect myogenesis and muscle growth and may not be a real atrophy. Moroever several compensation adaptation in the different tissue can have an impact on exercise performance. Do GSK knockout mice lose more muscle mass after denervation or starvation than wild type animals?

Point 6: The authors refer: ".....mice overexpressing Akt that develop muscular abnormalities around 6 months of age.....", the sentence is incorrect and should be adjusted. Indeed, the mice were Akt inducible and abnormalities happened when Akt was induced in adult mice and kept chronically active for 6 months. The alteration was due to Akt-mediated inhibition of autophagy-system. Is basal autophagy suppressed in SGK transgenic? Autophagy should be determined as above described (see comments in point 2, LC3 lipidation, LC3 puncta and p62 protein and mRNA). Quality of Figure 6A should be improved.

Point 7: Figure 7A, innervated fibers overexpressing SGK look bigger than controls. Indeed, it looks that there is an enrichment of fibers around 90-100 micron of diameter in caSGK transfected fibers (Supplementary Fig S6B) that result in a second peak in the distribution curve of fibers diameter.

Referee #2 (Comments on Novelty/Model System):

The global knockout and overexpression models are difficult to interpret and should be complemented by more detailed in vivo transfection experiments, as specified in the comments to the author.

Referee #2 (Other Remarks):

The kinase SGK is proposed to play an important role in the maintenance of muscle mass in hibernating animals and in preventing muscle atrophy in mice during starvation or limb immobilization. This conclusion, based on biochemical studies in hibernating squirrels and on the effect of Sgk1 knockout and overexpression in mice, is novel and interesting. However, several point are not convincing.

Major points

1. The level of SGK (SGK1?) and activated phospho-SGK, but not that of activated Akt, is increased in hibernating muscles, which do not undergo muscle atrophy in spite of immobility and starvation. In addition, SGK is also increased in dystrophic muscles of mdx mice and human DMD. The authors explain this finding with the hypertrophic response seen in muscular dystrophy. However, it appears that SGK is strongly expressed in mdx muscle fibers of varying size (Fig. 3C), including very small fibers, thus there is no relation with hypertrophy. This is confusing, muscular dystrophy is not an appropriate model of hypertrophy because of the coexistence of degenerating and regenerating fibers, denervated fibers, due to segmental necrosis events, and fibers undergoing compensatory hypertrophy. The authors should use well established models of muscle atrophy, such as denervation atrophy, and of muscle hypertrophy, such as functional hypertrophy induced by incapacitation or removal of synergistic muscles. The level of both phospho- and total SGK should be determined by Western blotting in these models at various time point during the atrophy and hypertrophy process.

2. Given the ubiquitous pattern of expression of SGK1 (Kobayashi et al, 1999), analysis of models involving global knockout or overexpression of Sgk1 are difficult to interpret due to possible secondary effects. Muscle-specific loss- and gain-of-function models would be required. The authors try to overcome this problem by using local electroporation-mediated gene transfer with kinase dead and constitutively active SGK mutants to define the role of SGK on muscle fiber size in control and immobilized muscles. However, this is the weakest part of the paper. Gene transfer

efficiency is not defined in these experiments and the biochemical effects of gene transfer on downstream effectors of SGK (mTORE and FOXO pathways) are not determined. This should be done.

Additional points

1. The title: "Activation of SGK is sufficient to maintain ..." appears to overstate the role of SGK.
2. Cross-sectional area should be used rather than min Feret diameter to evaluate muscle fiber size. In addition, perfectly transversal section should be used for these analyses: for example, Fig. S4D shows an obliquely sectioned Tg.Sgk1 muscle that would give false results in fiber size measurements.
3. Inspection of Fig. 7A seems to show an increased size of control fibers transfected with caSGK compared to fibers transfected with GFP, which is not seen in the quantitative measurements in Fig. 7B.
4. Are the antibodies to SGK used in WB and immunofluorescence (Fig. 3) specific for SGK1 or do they cross-react with other SGK isoforms? If they are specific, the term SGK1 should be used throughout instead of SGK.
5. SGK (SGK1?) is reported to be localized specifically in type IIB fibers. Thus one would expect that it is not present or to be poorly expressed in soleus muscle. Since SGK has not been characterized in skeletal muscle, it would be of interest to examine by WB SGK expression in fast and slow muscles.
6. The fact that hybernation is not accompanied by muscle atrophy is well established in different animal models, therefore Fig. 1 should be transferred to the Suppl Figs.

Referee #3 (Comments on Novelty/Model System):

Hibernating squirrel is a very interesting model to study.

Referee #3 (Other Remarks):

This is a very interesting study using hibernating squirrels to understand muscle atrophy. The authors demonstrate that serum- and glucocorticoid- inducible kinase (SGK) regulates muscle maintenance via down-regulation of proteolysis and autophagy, and increased protein synthesis during hibernation. In addition, the authors show that over expression of SGK protects against starvation and disuse muscle atrophy in non-hibernating mammals. Overall, this study provides a novel therapeutic target to prevent muscle atrophy.

This is an excellent and well written manuscript. The findings are novel and it should be of high interest in the field. My only suggestion would be for the authors to comment on fiber type distribution in Figures 1,4,5 and 6.

1st Revision - Authors' Response 14 August 2012

Referee #1:

The group of Cohn RD and coauthors is presenting an interest study about the mechanism that protects ground squirrel from atrophy during hibernation. The authors found that Serum and Glucocorticoid inducible Kinase (SGK) is activated during hibernation while Akt is not. The authors validate the involvement of SGK by using SGK knockout and SGK transgenic mice. SGK knockout mice show a decrease in myofiber size while the transgenic mice are protected from muscle atrophy. Activation of SGK inhibits the activation of the autophagy-lysosome and ubiquitin-proteasome proteolytic systems. The paper is of absolute interest for biologists, medical doctor and physiologists. Moreover, having found that SGK is an nticatabolic factor open the field to the development of new drugs to counteract muscle wasting and to further investigations on the mechanisms of these actions. However, the paper needs further experiments to clarify the mechanisms of SGK action and some controls. The following points should be considered by the authors.

We are very grateful for the overall positive critique about our manuscript and we have addressed the points raised below.

Point 1: The authors should better characterize the squirrel muscles. Is the proportion of beta-oxidative versus glycolitic fibers changed during hibernation? Are the level of PGC1a-PGC1b also affected by hibernation? This is an important aspect since b-oxidative fibers are known to be resistant to muscle atrophy.

We would like to thank the reviewer for this important comment. We have recently submitted a manuscript to PLoS One, which describes in detail that hibernating animals do not only demonstrate inhibition of muscle atrophy and autophagy mediated by SGK-1 as described here, but also increase mitochondrial metabolism. The paper is currently in revision, but we have accompanied the figures and figure legends of this manuscript.

Point 2: Figure 2, the characterization of the proteolytic system needs additional data. Is the total amount of ubiquitinated proteins changed in squirrel during hibernation? Despite the expression of the autophagy genes, authors should determine the level of LC3 lipidation (LC3II/LC3I ratio) and p62/SQSTM1 (protein/mRNA). Moreover, the amount of autophagosome revealed as LC3 positive puncta should be also determined. In the case that LC3 and p62 antibodies are not available for squirrel the authors might use electron microscopy to determine whether autophagy is present during hibernation. Moreover these analyses should be applied to muscles of SGK1 transgenic and knockout mice.

We have added a western for the total amount of ubiquitinated proteins in hibernating and non-hibernating squirrels to the supplementary figures demonstrating a significant increase in ubiquitinated proteins during hibernation. These results further strengthen our data that there is no evidence for increased proteolysis during hibernation in skeletal muscle (Fig. S1F).

Furthermore, we have added western blots of LC3B and p62 in hibernating and non-hibernating squirrels, demonstrating a decreased LC3II/LC3I ratio and increased expression of p62 levels in hibernating skeletal muscle (Fig. S1D and E). These findings demonstrate that autophagy is suppressed during hibernation. We have also performed LC3 staining in hibernating squirrels, which shows increased puncta staining (Fig. S1D). Both together our data suggest accumulation of autophagosomes supporting our data that autophagy is not increased in skeletal muscle during hibernation.
We have also performed western blot analyses of LC3B and p62 in *sgk1* knockout and transgenic animals. We have added the results to the supplementary figures (Fig. S3B, S4F and S5B). We did not find any significant difference in the expression of LC3B-II and p62 in wild-type versus *sgk1*$^{-/-}$ mice. Furthermore, we were not able to detect LC3B-I in this mice.
Interestingly, we did find increased levels of LC3B-II and p62 in steady state muscle of *sgk1* transgenic mice when compared to wild-type mice, which suggests that these mice may have mildly increased autophagy activity at baseline, no differences were found for LC3B-I. We are currently in the process of conducting a series of experiments to further analyze this interesting observation. The mild increase in autophagy at baseline suggests a more diverse role of SGK1 in the regulation of autophagy and could explain that *sgk1* transgenic mice do not develop any skeletal muscle abnormalities with increased age. LC3B puncta staining was performed but no differences were found between wild-type versus *sgk1*$^{-/-}$ and *sgk1* transgenic mice (given the amount of supplementary data, we have not included these images).

Point 3: The authors should quantify the phosphorylation status of Akt at threonin 308. This is important since recent findings underline a potential different role of the phopshorylation sites on Akt activity on downstream targets.

We have added the data to the manuscript (Fig. S1B). The phosphorylation status of Akt at threonine 308 is not altered between hibernating and non-hibernating squirrels.

Point 4: Figure 3C is unnecessary for the story and should be removed. Indeed the finding that total SGK is increased in dystrophin-deficient myofiber does not mean that SGK has a functional relevance. If the authors want to show these data they have to prove that SGK is more phosphorylated in hypertrophic fibers and cross the SGK transgenic with mdx mice and show amelioration. Personally, I do not think that the dystrophin deficient data fit with the story. Indeed the mechanisms that prevent muscle wasting during atrophy are different from the mechanisms that protect from degeneration. An additional minor point is related to the bibliography. The first paper that describes a beneficial effect of Akt on dystrophin-deficient muscles is Blaauw et al. 2008, Hum Mol Genet. and not Peter et al. 2009.

We agree with the reviewer that the data in *mdx* mice are unnecessary at this

point and we have removed the data from the manuscript.

Point 5: Figure 4A, the picture is of poor quality and it is difficult to determine the changes in morphology (small rounded. Are CSA of gastrocnemius and soleus muscles affected by the absence of GSK?

We have improved the quality of the H&E image in Figure 4A. Furthermore, we have performed morphometric analyses of gastrocnemius and soleus muscle in wild-type and *sgk1*$^{-/-}$ mice and added the data to the supplementary file (Fig. S3E). Our analyses demonstrate a decrease of muscle fiber size in gastrocnemius and soleus muscles in *sgk1*$^{-/-}$ mice.

The authors focused their attention on exercise alterations and force measurements that are indeed important aspects. However it would be of interest to know whether these mice are more prone to atrophy in catabolic conditions. In fact the classical knockout approach induce an early and systemic deletion of the studied gene. Therefore the decrease of CSA might be a consequence of a defect myogenesis and muscle growth and may not be a real atrophy. Moroever several compensation adaptation in the different tissue can have an impact on exercise performance. Do GSK knockout mice lose more muscle mass after denervation or starvation than wild type animals?

We would like to thank the reviewer for this very thoughtful comment. We have now performed immobilization and starvation experiments in the *sgk1*$^{-/-}$ mice. Strikingly, our results demonstrate a significantly exaggerated response of *sgk1*$^{-/-}$ mice to both, immobilization and starvation atrophy (Fig. S3F). These results further demonstrate that SGK1 is an important mediator of muscle mass and homeostasis in particular during catabolic and stressful situations such as immobilization and starvation.

Point 6: The authors refer: ".....mice overexpressing Akt that develop muscular abnormalities around 6 months of age.....", the sentence is incorrect and should be adjusted. Indeed, the mice were Akt inducible and abnormalities happened when Akt was induced in adult mice and kept chronically active for 6 months. The alteration was due to Akt-mediated inhibition of autophagy-system. Is basal autophagy suppressed in SGK transgenic? Autophagy should be determined as above described (see comments in point 2, LC3 lipidation, LC3 puncta and p62 protein and mRNA). Quality of Figure 6A should be improved.

We apologize for misrepresenting the previously published results and have changed the sentence accordingly. As mentioned above, we have performed analyses of LC3B and p62 in *sgk1* transgenic mice and found evidence for slightly increased basal levels of autophagy. We have started a series of experiments to address this question in more detail, but we feel that these comprehensive analyses are beyond the scope of the current manuscript. Figure 6A has been improved.

Point 7: Figure 7A, innervated fibers overexpressing SGK look bigger than controls. Indeed, it looks that there is an enrichment of fibers around 90-100 micron of diameter in caSGK transfected fibers (Supplementary Fig S6B) that result in a second peak in the distribution curve of fibers diameter.

We agree with the reviewer that there is a visual impression that muscle fibers overexpressing SGK1 are larger. While the trend for increased muscle fibers certainly exists, we were not able to demonstrate statistical significance for this observation (ANOVA p value is 0.0977).

Referee #2 (Comments on Novelty/Model System):

The global knockout and overexpression models are difficult to interpret and should be complemented by more detailed in vivo transfection experiments, as specified in the comments to the author.

Referee #2 (Other Remarks):

The kinase SGK is proposed to play an important role in the maintenance of muscle mass in hibernating animals and in preventing muscle atrophy in mice during starvation or limb immobilization. This conclusion, based on biochemical studies in hibernating squirrels and on the effect of Sgk1 knockout and overexpression in mice, is novel and interesting. However, several point are not convincing.

We would like to thank the reviewer for the overall positive assessment of our paper.

Major points

1. The level of SGK (SGK1?) and activated phospho-SGK, but not that of activated Akt, is increased in hibernating muscles, which do not undergo muscle atrophy in spite of immobility and starvation. In addition, SGK is also increased in dystrophic muscles of mdx mice and human DMD. The authors explain this finding with the hypertrophic response seen in muscular dystrophy. However, it appears that SGK is strongly expressed in mdx muscle fibers of varying size (Fig. 3C), including very small fibers, thus there is no relation with hypertrophy. This is confusing, muscular dystrophy is not an appropriate model of hypertrophy because of the coexistence of degenerating and regenerating fibers, denervated fibers, due to segmental necrosis events, and fibers undergoing compensatory hypertrophy. The authors should use well established models of muscle atrophy, such as denervation atrophy, and of muscle hypertrophy, such as functional hypertrophy induced by incapacitation or removal of synergistic muscles.

We absolutely agree with the reviewer that the *mdx* mouse model is not an adequate model for hypertrophy. We have removed this figure from the revised version of the manuscript.

We have not used denervation as a model to induce muscle atrophy since data from our laboratory suggest that the molecular mechanisms underlying denervation atrophy are inherently different from the catabolic aspects of muscle atrophy during immobilization and/or starvation. We are currently in the process of preparing a manuscript that will detail our findings. Briefly, when we used mice that underwent denervation and immobilization and exposed these to myostatin inhibition via administration of ACTIIB soluble receptor, we found that only immobilization atrophy was prevented by ACTIIB but NOT denervation atrophy. Further molecular analyses in treated and non-treated mice revealed that the Akt/SGK1/FOXO/mTOR pathway behaves significantly different in denervation muscles as compared to muscles undergoing immobilization. Thus, given that the main conditions of hibernating animals evolve around immobilization and prolonged starvation, we have focused our analyses of SGK1 on these catabolic circumstances in *sgk1$^{-/-}$ and transgenic* mice.

We performed analyses of SGK1 expression in immobilized mice and IGF-1 overexpressing mice that develop skeletal muscle hypertrophy of type II fibers. Our results demonstrate an increased expression of SGK1 in hypertrophic fibers of IGF-1 transgenic mice (Fig. S2C). Interestingly, the expression pattern of SGK1 in mice undergoing immobilization atrophy exhibits a dynamic pattern at various different time points. Our results show that SGK1 expression is initially increased after 3 days of immobilization and then decreased at 7 days followed by increased expression of SGK1 at 10, 14 and 21 days (Please see enclosed figure).

We are currently in the process of analyzing the dynamic expression pattern of additional members of the IGF-1/Akt/Foxo/mTOR pathway during various time points of immobilization atrophy. Our preliminary data suggest that proteins involved in protection against muscle atrophy behave different from proteins involved in protein synthesis.

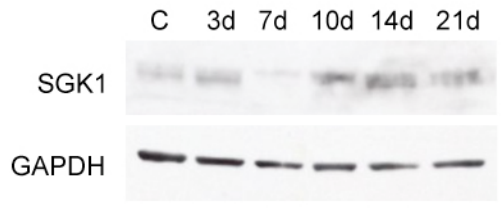

SGK1 expression in immobilized mice at different time time points. The SGK1 expression in mice undergoing immobilization shows that SGK1 is initially increased after 3 days of immobilization and then decreased at 7 days followed by increased expression of SGK1 at 10, 14 and 21 days

The level of both phospho- and total SGK should be determined by Western blotting in these models at various time point during the atrophy and hypertrophy process.

Unfortunately, we were not able to test the expression levels of phosphorylated SGK1 T256 since the antibody from Cell Signalling is not longer available. More antibodies from different companies have been tested in transfected cells and tissue without success. We are in the process of producing our own antibody. However, we would like to point out that in contrast to Akt, SGK1 is

transcriptionally regulated, thus expression of total SGK1 can be used as an indicator of increased SGK1 activity.

Lastly, we have changed our nomenclature to SGK1 throughout the manuscript.

2. Given the ubiquitous pattern of expression of SGK1 (Kobayashi et al, 1999), analysis of models involving global knockout or overexpression of Sgk1 are difficult to interpret due to possible secondary effects. Muscle-specific loss- and gain-of-function models would be required. The authors try to overcome this problem by using local electroporation-mediated gene transfer with kinase dead and constitutively active SGK mutants to define the role of SGK on muscle fiber size in control and immobilized muscles. However, this is the weakest part of the paper. Gene transfer efficiency is not defined in these experiments and the biochemical effects of gene transfer on downstream effectors of SGK (mTORE and FOXO pathways) are not determined. This should be done.

We would like to thank the reviewer for this thoughtful comment. We have now included analyses of the gene transfer efficiency and have added these data to the supplementary figures and materials (Fig. S6D).
Furthermore, we have conducted expression analyses of Foxo3a and members of the mTOR pathway (Fig. S6C). Our results demonstrate an increased phosphorylation of Foxo3a and p-4EBP1 in muscles overexpressing caSGK1. These results further support our observation that immobilized muscles overexpressing SGK1 are protected against muscle atrophy.

Additional points

1. The title: "Activation of SGK is sufficient to maintain ..." appears to overstate the role of SGK.

   We have changed the title accordingly to Activation of SGK is important to maintain skeletal muscle homeostasis and prevent muscle atrophy.

2. Cross-sectional area should be used rather than min Feret diameter to evaluate muscle fiber size. In addition, perfectly transversal section should be used for these analyses: for example, Fig. S4D shows an obliquely sectioned Tg.Sgk1 muscle that would give false results in fiber size measurements.

   While cross-sectional area is commonly used in the literature for morphometric analyses in skeletal muscle, we have purposefully used the minimal Feret's diameter to express muscle fiber size. In a very thorough study by Briguet et al., Neuromuscul Disord 2004 it was established that the minimal Feret's diameter is the most robust parameter that particularly minimizes experimental errors such as orientation of the sectioning angle.

3. Inspection of Fig. 7A seems to show an increased size of control fibers transfected with caSGK compared to fibers transfected with GFP, which is not

seen in the quantitative measurements in Fig. 7B.

While we observe a trend of larger muscle fibers in mice transfected with caSGK1, we were not able to establish statistical significance for this trend (ANOVA p value is 0.0977).

4. Are the antibodies to SGK used in WB and immunofluorescence (Fig. 3) specific for SGK1 or do they cross-react with other SGK isoforms? If they are specific, the term SGK1 should be used throughout instead of SGK.
5. SGK (SGK1?) is reported to be localized specifically in type IIB fibers. Thus one would expect that it is not present or to be poorly expressed in soleus muscle. Since SGK has not been characterized in skeletal muscle, it would be of interest to examine by WB SGK expression in fast and slow muscles.

We would like to thank the reviewer for this comment. The antibodies are indeed specific for SGK1 and have changed the term throughout the manuscript accordingly.

Moreover, we have performed expression analyses of SGK1 in various muscles (Fig. S2B). Indeed, we find that SGK1 is most highly expressed in tibialis anterior muscle, heart and diaphragm, with slightly lower levels of SGK1 protein expression in the mixed fiber type extremity muscles of quadriceps and gastrocnemius and even lower expression in mainly type II soleus muscle.

6. The fact that hybernation is not accompanied by muscle atrophy is well established in different animal models, therefore Fig. 1 should be transferred to the Suppl Figs.

We certainly agree with the reviewer that protection of skeletal muscle against atrophy is a know phenomenon in hibernating animals. The main reason why we decided to present this figure is that previously published manuscripts have mostly analyzed smaller muscles (EDL, EDB etc.) and did not show morphological integrity of skeletal muscle fibers during hibernation. However, we would of course be happy to remove this figure from the main body of the manuscript, if the reviewer feels very strongly about this.

Referee #3 (Comments on Novelty/Model System):

Hibernating squirrel is a very interesting model to study.

Referee #3 (Other Remarks):

This is a very interesting study using hibernating squirrels to understand muscle atrophy. The authors demonstrate that serum- and glucocorticoid- inducible kinase (SGK) regulates muscle maintenance via down-regulation of proteolysis and autophagy, and increased protein synthesis during hibernation. In addition, the authors show that

over expression of SGK protects against starvation and disuse muscle atrophy in non-hibernating mammals. Overall, this study provides a novel therapeutic target to prevent muscle atrophy.

This is an excellent and well written manuscript. The findings are novel and it should be of high interest in the field. My only suggestion would be for the authors to comment on fiber type distribution in Figures 1,4,5 and 6.

We would like to thank this reviewer for the wonderful and positive evaluation of our manuscript. We have added comments to the fiber type distribution of figures 4,5 and 6. We did not comment about the fiber type distribution of figure 1 as we have a manuscript in revision at PLoS One that describes increased mitochondrial metabolism in skeletal muscle of hibernating animals, which is in part due to an increase in type I muscle fibers. The paper is currently under revision, but we have accompanied the figures and figure legends of this manuscript.

2nd Editorial Decision 10 September 2012

Thank you for the submission of your revised manuscript "Activation of Serum/Glucocorticoid-Induced Kinase 1 (SGK1) is important to maintain skeletal muscle homeostasis and prevent atrophy" to EMBO Molecular Medicine. We have now received the enclosed reports from the referees whom we asked to re-assess it.

As you will see, the Reviewers acknowledge that the manuscript was significantly improved during revision and Reviewer #1 indicates that it is suitable for publication. However, Reviewer #2 still raises concerns that should be convincingly addressed. Since we do acknowledge the potential interest of your findings, we would be willing to consider a revised manuscript with the understanding that the referee concerns must be convincingly and conclusively addressed.

Specifically, the Reviewer highlights that the new hypertrophy model should be investigated in some more depth and that SGK1 activation should be demonstrated.

Revised manuscripts should be submitted latest within three months of a request for revision; they will otherwise be treated as new submissions, unless arranged otherwise with the editor.

I look forward to seeing a revised form of your manuscript as soon as possible.

Yours sincerely,

Editor
EMBO Molecular Medicine

***** Reviewer's comments *****

Referee #1:

This reviewer is satisfied by the revised version. The authors addressed all my concerns.

Referee #2 (Comments on Novelty/Model System):

The new mIGF1 model presented in the revised version is not described in the Methods, and is dealt with superficially.

Referee #2 (Other Remarks):

While the revised manuscript is improved compared to the original version, some of the criticisms raised to the original version have not been addressed, and some additional data presented are not clear.

Major point 1. HYPERTROPHY MODELS. The authors agree that muscular dystrophy (mdx mouse) is not a good model of muscle hypertrophy and have therefore deleted these results, however they present only superficial data (a short paragraph, page 8) on another hypertrophy model, namely transgenic mice that over-express IGF-1 in skeletal muscle. This mIgf-1 model is not referred to in the Methods. In mIgf-1 muscles the authors report "increased SGK1 expression in type IIB hypertrophic muscle fibers, suggesting that SGK1 mediates muscle hypertrophy in mIgf-1 mice (Fig. S2C)." However, this is not demonstrated in the figure since a serial section stained for myosin IIB is missing and quantitative data on fiber size in relation with fiber type are not presented. Furthermore, a role of SGK1 in mediating hypertrophy should be established by interfering with SGK1.

ANTI-PHOSPHO SGK1 ANTIBODIES. The problems encountered with anti-phospho SGK1 are surprising, because such antibodies are currently used with good results in several published studies (e.g. Fang Z et al, J Biol Chem. 2012 Jan 13;287(3):2090-8. Epub 2011 Dec 2; Baskin R, Sayeski PP. Cell Signal. 2012 Feb;24(2):435-42. Epub 2011 Sep 22.). The central point of this paper, as stated in the title, is that "Activation of Serum/Glucocorticoid-Induced Kinase 1 (SGK1) is important to maintain skeletal muscle homeostasis and prevent atrophy", therefore the demonstration of SGK1 activation in this work is imperative.

Additional point 5. FIBER TYPES. The immunoblotting findings on SGK1 distribution in different muscles (Fig. S2B) are surprising in view of the specific localization of SGK1 by immunofluorescence in type IIB fibers (Fig. S2A). In fact, SGK1 antibodies give a strong signal in the mouse diaphragm, which is known to contain only rare type IIB fibers, but a weak signal in the quadriceps, which contains many type IIB fibers. The question of fiber types is further complicated by the additional results from the other paper submitted to PLoS One, in which it is shown that type IIB fibers are dramatically decreased in the hybernating squirrel, in which SGK1 is activated, whereas no change in fiber type composition is induced by overexpressing caSGK1. These discrepancies should be clarified.

2nd Revision - Authors' Response 25 September 2012

Referee #2 (Comments on Novelty/Model System):

The new mIGF1 model presented in the revised version is not described in the Methods, and is dealt with superficially.

Referee #2 (Remarks):

While the revised manuscript is improved compared to the original version, some of the criticisms raised to the original version have not been addressed, and some additional data presented are not clear.

We are very grateful for the overall positive critique about our manuscript and we have addressed the major points raised below.

Major point 1. HYPERTROPHY MODELS. The authors agree that muscular dystrophy (mdx mouse) is not a good model of muscle hypertrophy and have therefore deleted these results, however they present only superficial data (a short paragraph, page 8) on another hypertrophy model, namely transgenic mice that over-express IGF-1 in skeletal muscle. This mIgf-1 model is not referred to in the Methods. In mIgf-1 muscles the authors report "increased SGK1 expression in type IIB hypertrophic muscle fibers, suggesting that SGK1 mediates muscle hypertrophy in mIgf-1 mice (Fig. S2C)." However, this is not demonstrated in the figure since a serial section stained for myosin IIB is missing and quantitative data on fiber size in relation with fiber type are not presented.

We would like to thank the reviewer for this important comment. A detailed characterization of the skeletal muscle of IGF-1 transgenic mice was initially presented by Musaro et al., 2001. In this study, the authors performed quantification of fiber size in relation with fiber type demonstrating hypertrophy of type IIB muscle fibers. We have included this reference in the manuscript and the methods. Furthermore, we have repeated and confirmed these studies and have included in Fig. S2E.

To further support the fact that SGK1 is increased in type IIB muscle fibers, we have now included serial sections stained for fiber type IIB and SGK1 concomitantly (Fig. S2E).

Furthermore, a role of SGK1 in mediating hypertrophy should be established by interfering with SGK1.

We very much appreciate this suggestion. However, our preliminary experiments demonstrate that in vivo knock-down of SGK1 expression in wild-type mice postnatally induces a compensatory increase of Akt without any phenotypical short-term

impact (10 days after SGK1 knock-down) on skeletal muscle morphometry and function. We are currently in the process of following up on these observations mostly by extending our observation period. However, these experiments carry the potential for a lot of new and important scientific data and will likely open up novel scientific avenues. Thus, we feel that these experiments are beyond the scope of this manuscript.
We have included a brief statement about this issue in our discussion.

We feel that we have added a number of additional data to the revised version of the manuscript. We have now demonstrated that the absence of SGK1 in skeletal muscle leads to an exaggerated response to disuse and starvation atrophy, despite a compensatory increase in Akt.
Furthermore, our electroporation experiments are now providing phenotypic and mechanistic evidence that increased expression of activated SGK1 in vivo protects against disuse muscle atrophy. Taken together, we hope that the reviewer agrees that we have provided substantial evidence that SGK1 plays a critical role in skeletal muscle homeostasis in vivo.

ANTI-PHOSPHO SGK1 ANTIBODIES. The problems encountered with anti-phospho SGK1 are surprising, because such antibodies are currently used with good results in several published studies (e.g. Fang Z et al, J Biol Chem. 2012 Jan 13;287(3):2090-8. Epub 2011 Dec 2; Baskin R, Sayeski PP. Cell Signal. 2012 Feb;24(2):435-42. Epub 2011 Sep 22.). The central point of this paper, as stated in the title, is that "Activation of Serum/Glucocorticoid-Induced Kinase 1 (SGK1) is important to maintain skeletal muscle homeostasis and prevent atrophy", therefore the demonstration of SGK1 activation in this work is imperative.

We agree with the reviewer that the phosphorylation status of SGK1 is essential for this work. We thank the reviewer for the antibodies suggestions.
We have added the data to the manuscript (Fig. S2D). The phosphorylation status of SGK1 is increased in mIgf-1 transgenic mice. Furthermore, we have performed western blot analyses of total and phosphorylated AKT, which is not altered in mIGF-1 transgenic mice as previously reported (Fig. S2D).

Additional point 5. FIBER TYPES. The immunoblotting findings on SGK1 distribution in different muscles (Fig. S2B) are surprising in view of the specific localization of SGK1 by immunofluorescence in type IIB fibers (Fig. S2A). In fact, SGK1 antibodies give a strong signal in the mouse diaphragm, which is known to contain only rare type IIB fibers, but a weak signal in the quadriceps, which contains many type IIB fibers. The question of fiber types is further complicated by the additional results from the other paper submitted to PLoS One, in which it is shown that type IIB fibers are dramatically decreased in the hybernating squirrel, in which SGK1 is activated, whereas no change in fiber type composition is induced by overexpressing

caSGK1. These discrepancies should be clarified.

Due to the length of the manuscript we only included the staining of fiber type IIB as a representative staining, but we detected low levels of SGK1 expression in other type II fibers, in particular type IIA. Approximately 61% of diaphragm muscle fibers are composed of type II fibers, which likely explains the strong signal. We have changed the text in the manuscript accordingly.

Although we use the hibernating squirrel model as the initial tool to identify SGK1 as modulator of muscle homeostasis, the underlying mechanisms of protection against muscle atrophy despite prolonged periods of immobilization and starvation are far more complex and involve a number of fine tuned biological systems and pathways. In order to overcome low blood flow and long-term hypoxia, changes in glucose and fat metabolism, and mitochondrial catabolism contribute to the animal survival and protection of skeletal muscle atrophy. We currently have a paper under review in PLoS ONE showing that in addition to the data showed in this paper and the swift towards a slow fiber composition, these phenotypic changes were also accompanied by an activation of the endurance pathway modulated by PGC1alpha with enhanced oxidative capacity. However, while there is an increase in type I muscle fibers (from 7-25%), the predominant muscle fiber type is still type II, with over 50% composed of type IIB muscle fibers.
