# Supplementary material for: Activation of serum/glucocorticoid-induced kinase 1 (SGK1) is important to maintain skeletal muscle homeostasis and prevent atrophy
Source: EMBO Mol Med. 2012 Nov 19;5(1):80–91. doi: 10.1002/emmm.201201443 (PMC3569655; doi:10.1002/emmm.201201443)
Supplement: Supplementary file 2 [file emmm0005-0080-SD2.pdf]

TABLE OF CONTENTS

Table S1	2
Table S2	3
Suppl Figure Legends	4
Figure S1	7
Figure S2	8
Figure S3	9
Figure S4	10
Figure S5	11
Figure S6	12

SUPPLEMENTARY INFORMATION

Table S1. Physical performance of SGK1 knockout mice (*sgk1*$^{-/-}$) and wild type littermates (*sgk1*$^{+/+}$) in running wheels during a 36-day period. Arithmetic means ± SD (n = 6 each), * indicates statistically significant ($p<0.01$) difference to *sgk1*$^{+/+}$ mice.

	sgk1$^{+/+}$ mice	*sgk1*$^{-/-}$ mice
Average speed (km/h)	1.35 ± 0.10	1.30 ± 0.09
Maximal speed (km/h)	4.13 ± 0.07	3.79 ± 0.19
Running distance (km/24h)	6.74 ± 0.74	4.08 ± 0.31*

Table S2. Statistical analysis of western blot quantification.

Protein	Mean±SD	n	Statistical Test
**13-lined ground squirrels**			
pAkt S478/Akt	S: 1.33±0.24<br>H: 0.69±0.19	n=7	t-test (p=0.002)
pFOXO3a S253/FOXO3a	S: 2.21±0.61<br>H: 4.69±1.67	n=7	t-test (p=0.013)
pP70S6K/P70S6K	S: 2.55±0.18<br>H: 4.34±0.79	n=9-10	t-test (p=0.001)
SGK/GAPDH	S: 0.34±0.05<br>H: 0.95±0.3	n=8	t-test (p=0.001)
pSGK T256/GAPDH	S: 0.81±0.43<br>H: 1.71±0.47	n=8	t-test (p=0.006)
pFOXO3A S315/GAPDH	S: 0.11±0.03<br>H: 0.27±0.18	n=7	t-test (p=0.029)
p62/GAPDH	S: 0.38±0.22<br>H: 0.75±0.18	n=7	t-test (p=0.016)
LC3B-II/LC3B-I	S: 0.44±0.18<br>H: 0.22±0.07	n=7	t-test (p=0.039)
Ubiquitin/GAPDH	S: 8.44±0.17<br>H: 12.25±2.18	n=7	t-test (p=0.022)
**SGK1 knockout mouse**			
pAkt/Akt	WT: 0.43±0.06<br>KO: 0.54±0.06	n=5	t-test (p=0.045)
**SGK1 transgenic mouse**			
pP70S6K/P70S6K	WT: 2.46±0.65<br>TG: 3.66±0.42	n=4	t-test (p=0.021)
p4EBP1/4EBP1	WT: 0.34±0.14<br>TG: 0.6±0.06	n=4	t-test (p=0.043)
pFOXO3a S253/FOXO3a	WT: 12.15±4.45<br>TG: 14.27±4.33	n=4	t-test (p=0.52)
pFOXO3a S315/FOXO3a	WT: 3.8±0.86<br>TG: 6.34±1.27	n=4	t-test (p=0.016)
pFOXO3a T32/FOXO3a	WT: 0.65±0.16<br>TG: 1.2±0.16	n=4	t-test (p=0.003)
p62/GAPDH	WT: 1.39±0.37<br>TG: 2.72±0.79	n=4	t-test (p=0.046)
LC3B-II/GAPDH	WT: 0.17±0.04<br>TG: 0.38±0.09	n=4	t-test (p=0.011)
**SGK1 transgenic mouse-starvation**			
pFOXO3a S253/FOXO3a	WT: 8.1±3<br>TG: 7.1±1.25	n=4	t-test (p=0.56)
pFOXO3a S315/FOXO3a	WT: 3.5±0.7<br>TG: 7.8±2.3	n=4	t-test (p=0.011)
pFOXO3a T32/FOXO3a	WT: 1.16±0.09<br>TG: 2.03±0.45	n=4	t-test (p=0.009)
Beclin/GAPDH	WT: 0.14±0.036<br>TG: 0.08±0.004	n=4	t-test (p=0.043)
LC3B/GAPDH	WT: 0.16±0.016<br>TG: 0.08±0.03	n=4	t-test (p=0.023)

SUPPLEMENTAL FIGURE LEGENDS

Fig. S1. A Percentage distribution of minimal Feret's diameter in quadriceps and tibialis anterior muscles is not significantly different between summer and hibernation. **B** Densitometric analyses from non-hibernating and hibernating squirrels demonstrates no significant difference in of Akt phosphorylation in T308. **C** Proteasome activity at 37°C indicates no loss of euthermic capacity during hibernation. The proteasome inhibitor, Lactacystin, was included as test inhibitor for control purpose. **D and E** Western blot analyses and densitometry show significant upregulation in p62 and a decrease of LC3B-II/LC3B-I ratio in hibernating squirrels. An increase of autophagosome (detected by immunostaining of LC3B puncta) is observed during hibernation. **F** Accumulation of ubiquitinated proteins is detected in hibernating animals.

Fig. S2. A Serial sections of skeletal muscle from hibernating squirrels stained for SGK1 and phosphorylated Foxo3a (P-Foxo3a) demonstrates co-localization in type II muscle fibers. Representative selection of muscle fibers co-expressing SGK and P-Foxo3a are indicated by asterisks (*). **B** SGK1 expression in different muscles. **C** Increased SGK1 expression in hypertrophic, type IIB fibers of *mIgf-1* transgenic muscles. **D** Western blot analyses show no significant changes in p-Akt and upregulation in phospho-SGK1 in *mIgf-1* muscles. **E** Quantification of the minimum Feret's diameter by fiber type of skeletal muscle from *mIgf-1* transgenic and WT mice. Representative serial sections of skeletal muscle from *mIgf-1* transgenic mice stained for SGK1 and type IIB muscle fibers. **F** Average body weight of wild-type and $sgk1^{-/-}$ mice is not significantly different, muscle weight to body weight ratio of tibialis anterior muscle is significantly lower in $sgk1^{-/-}$ mice when compared to wild-type mice (p=0.03 and p=0.04, respectively). **G** Fiber type distribution is not different in tibialis anterior muscles of wild-type and $sgk1^{-/-}$ mice.

Fig. S3. A Corresponding densitometry as a function of total Akt level for P-Akt (n=4 each group). **B** Western blots of *tibialis anterior* muscle from WT and $sgk1^{-/-}$ mice using

antibodies against the proteins indicated. **C** Representative recordings of isometric twitch contractions of soleus muscles in response to single supramaximal electrical stimuli (arrows). **D** Characteristics of twitch contractions of soleus muscles from wild-type (WT) and *sgk1*$^{-/-}$ mice: amplitudes, time to peak and half relaxation times of twitches as shown in C (p=0.01). **E** Percentage distribution and mean minimum Feret's diameter in gastrocnemius and soleus muscle (p=0.0037, p=0.0028). **F** Immobilization and starvation experiments in the *sgk1*$^{-/-}$ mice. There is a significant exaggerated response of *sgk1*$^{-/-}$ mice to immobilization (*p<0.002, #p<0.001 and §p<0.0001) and starvation (*p<0.00001, **p<0.0003 and #p<0.002) induced atrophy. **G** Western blot analyses of muscles from wild-type and *akt1*$^{-/-}$ mice demonstrate no alterations in phosphorylation levels of Foxo3a at serine 253, accompanied by an increase in SGK abundance and phosphorylation of S315 Foxo3a.

Fig. S4. **A** Total RNA extracted from *tibialis anterior* samples from control (WT) or transgenic (Tg) mice with the RNeasy mini spin system (GE Healthcare) was used as a template to produce single-stranded cDNA using a commercial kit (iScript cDNA synthesis kit, Biorad). Endogenous and transgenic SGK1 were simultaneously detected by RT-PCR using primers 5'- GGAAGCAGCAGAAGCCTTCCTCGG-3' and 5'-GACTGCCAAGCTTCCAGGTGTGC-3', which flank the stop codon and produce a 186 bp product with wild-type SGK1 and a 267 bp product with Tg.SGK1 due to the insertion of three consecutive copies of the HA-epitope before the stop codon. **B** Quantitative PCR analysis of total (endogenous plus transgenic) SGK1 expression in *tibialis anterior*. cDNA from control or transgenic animals was used as template for qPCR using primers 5'-CGGTTTCACTGCTCCCCTCAGTC-3' and 5'-GCGATGAGAATCGCTACCATTCCC-3', which amplify a 130 bp product common to both the control and the transgenic mRNA. A mouse GAPDH amplicon was used as housekeeping standard. Data points represent the average ± SD of three independent reactions (sample was run in triplicates

and average in each reaction, n=4 per genotype). **C** Morphometric analysis shows a decrease of muscle fiber size of the wild-type control mice compared with *sgk1*^{tg} transgenic littermates during starvation (p=0.0015). **D** Hematoxylin-eosin staining of *tibialis anterior* sections from 6-month old control and transgenic mice. Preparations were mounted using Eukitt mounting medium (Kindler, Freiburg, Germany). Images were obtained under a Leica DMR photomicroscope (Leica Microsystems) and compiled using Adobe Illustrator (Adobe Systems). Representative images from control and transgenic samples are shown, scale bar is 90 μm. **E** No switches in fiber type composition of wild-type and *sgk1*^{tg} mice in tibialis anterior and gastrocnemius muscles. **F** Western blot and densitometry analyses of muscles from wild-type and *sgk1*^{tg} mice demonstrate an increase of p62 and LC3B-II in *sgk1*^{tg}.

Fig. S5. A Gene expression levels of the atrogenes atrogin-1 and MuRF1 and autophagy marker MAP-1/LC3B. (for atrogin-1: *p < 0.05, #p<0.001, †p<0.001; for MURF1: #p<0.01, †p<0.01; for MAP1/LC3B: #p<0.01 using ANOVA). **B** Decreased protein abundance of Beclin, ATG7, LC3B in *sgk1*^{tg} mice after 48 hours of starvation .

Fig. S6. A Transfection of wild-type *Sgk1* (WT) and kinase dead *Sgk1* (KD) into immobilized tibialis anterior muscles (green, cytoplasmic staining) reveals decreased fiber size diameter when compared to control, non- immobilized tibialis anterior muscles (100 μm). Laminin γ-1 staining (red) outlines the basement membrane and blue staining marks nuclei (DAPI). **B** Representation of percentage distribution of the minimal Feret's Diameter of tibialis anterior muscle (non-immobilized) transfected with EGFP only (GFP), wild-type *Sgk1* (WT), kinase dead *Sgk1* (KD) and constitutively active *Sgk1* (CA). **C** Western blots of electroporated *tibialis anterior* using antibodies against the proteins indicated. **D** Gene transfer efficient calculated as percentage of EGFP positive fibers of the total cross-sectional area.

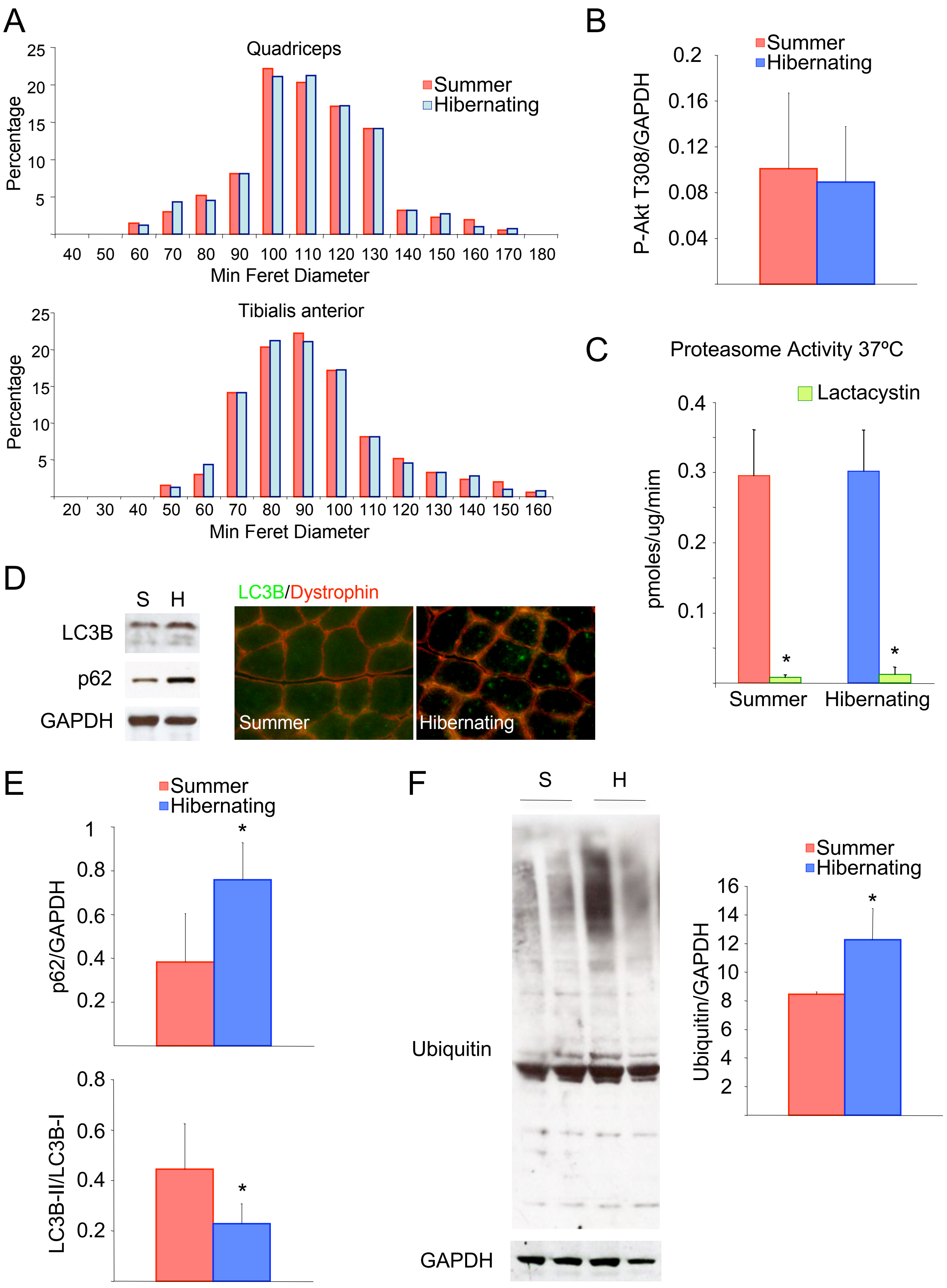

Figure-S1 (Cohn)

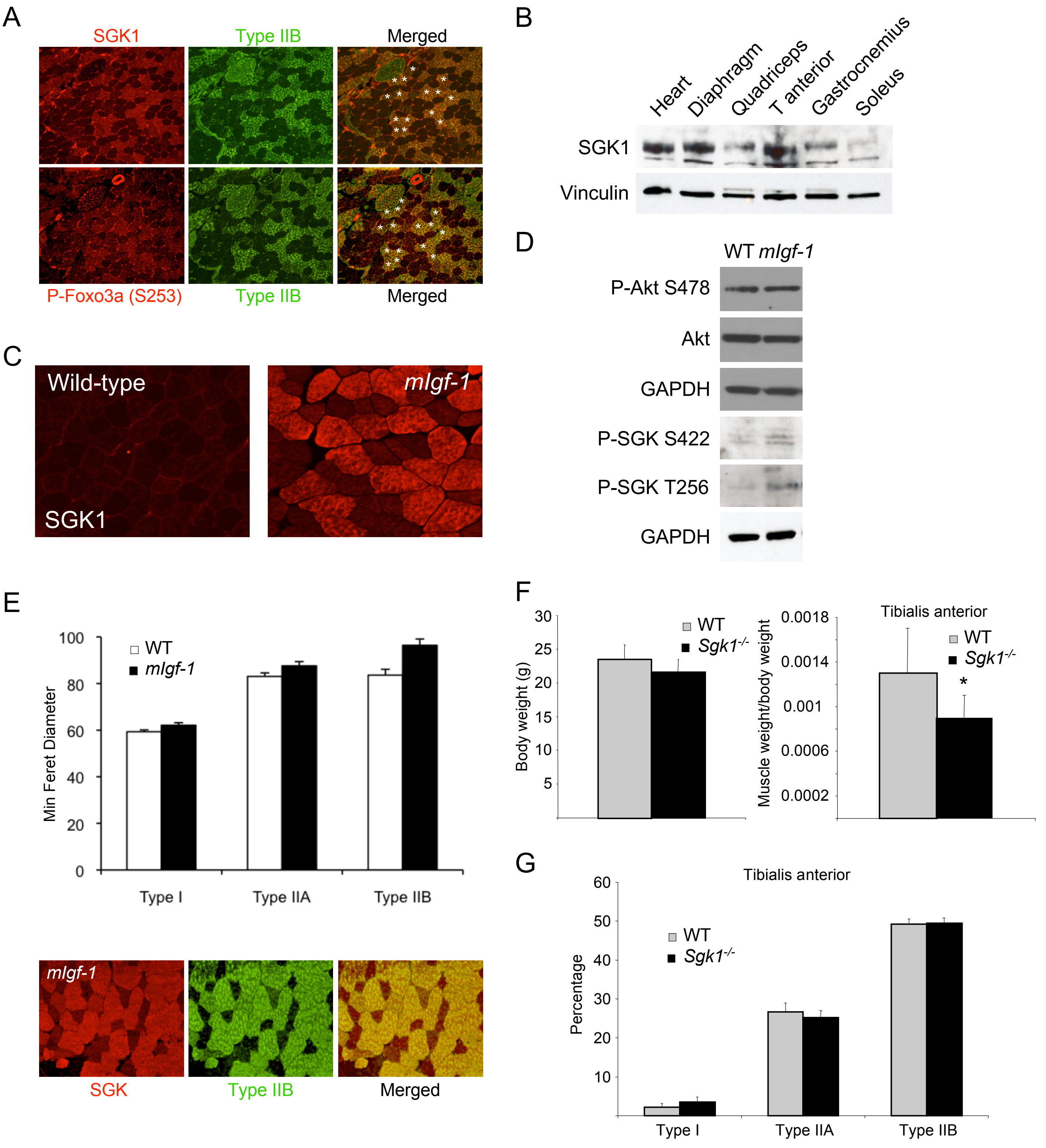

Figure-S2 (Cohn)

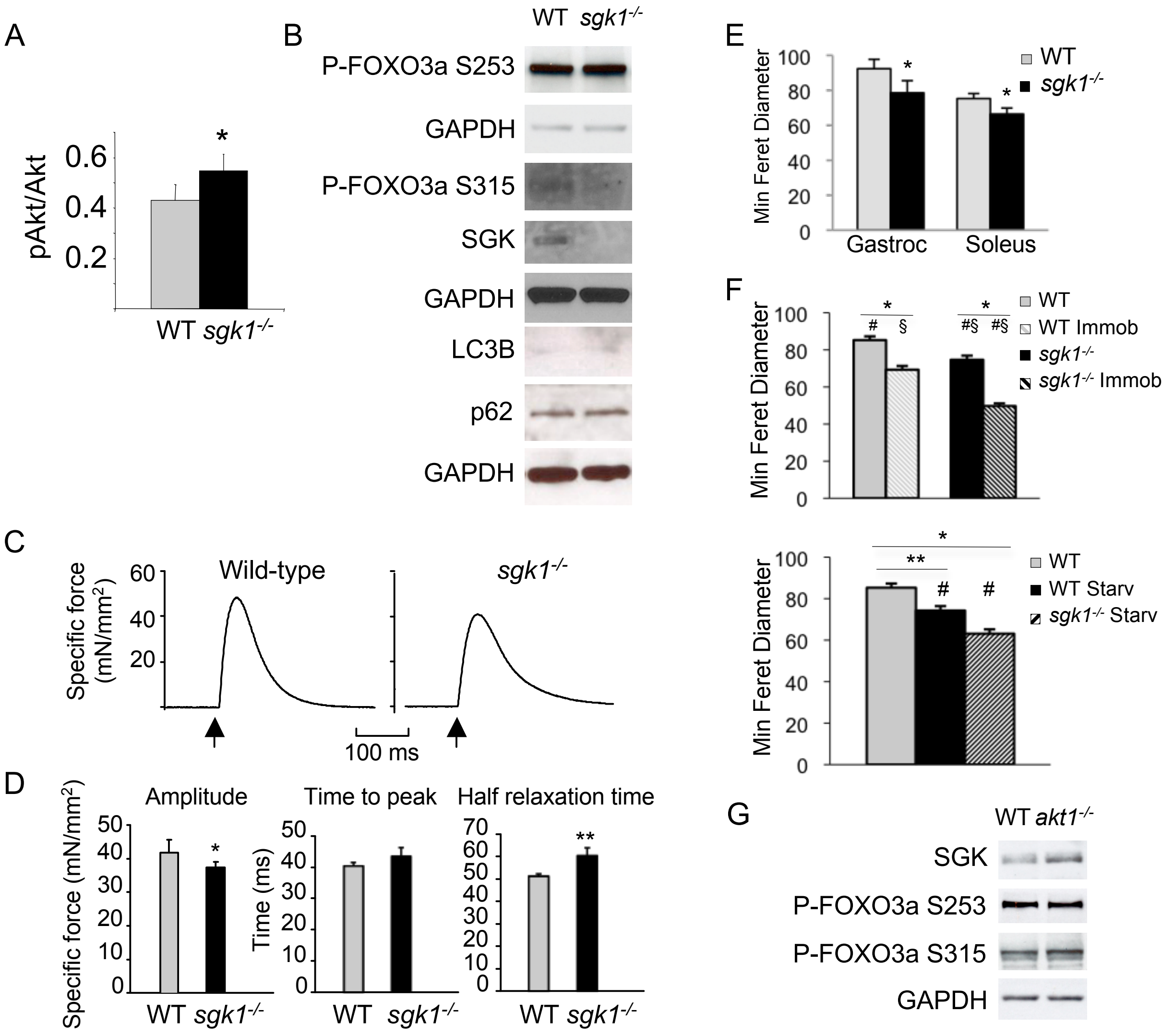

Figure-S3 (Cohn)

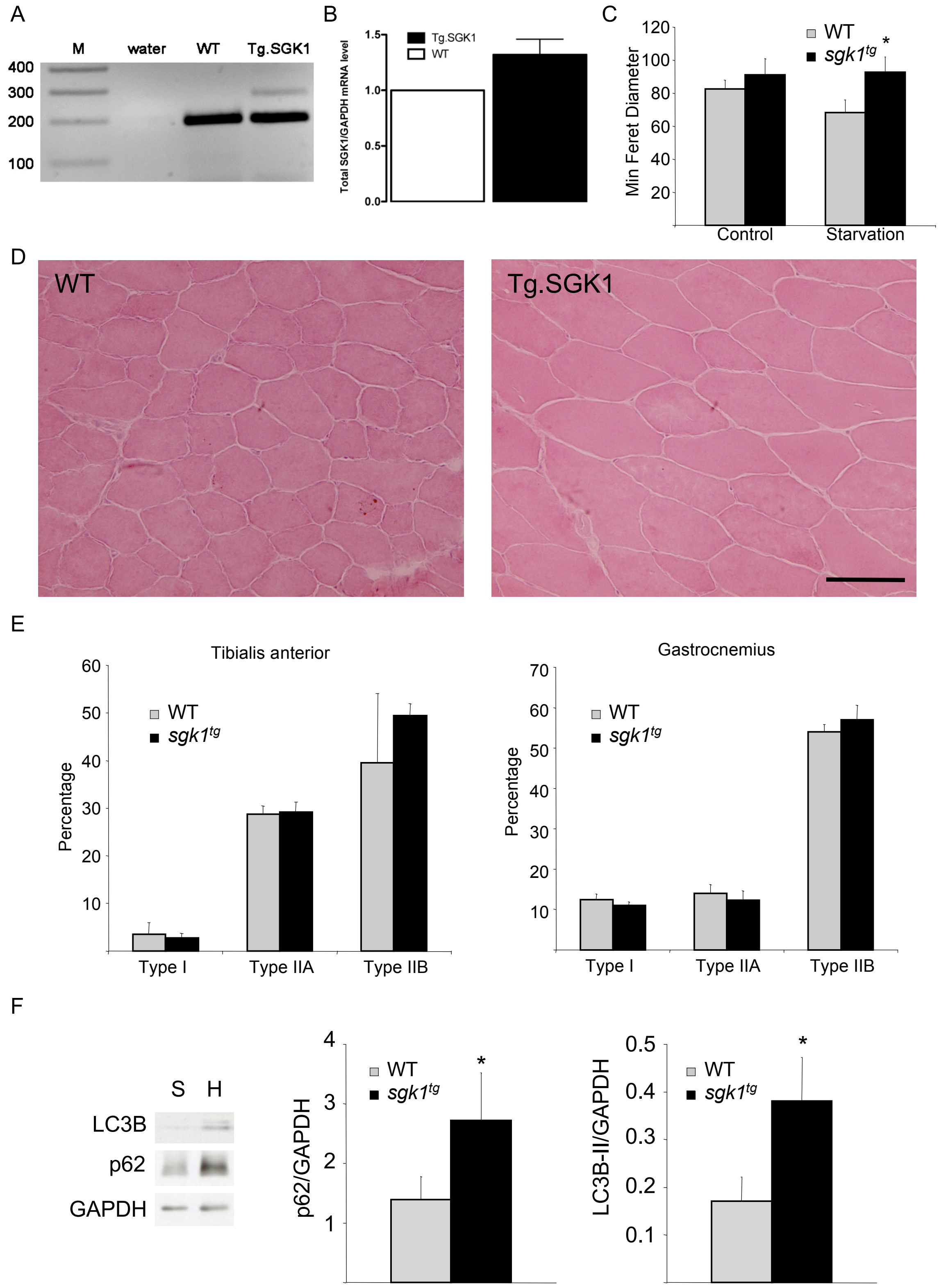

Figure-S4 (Cohn)

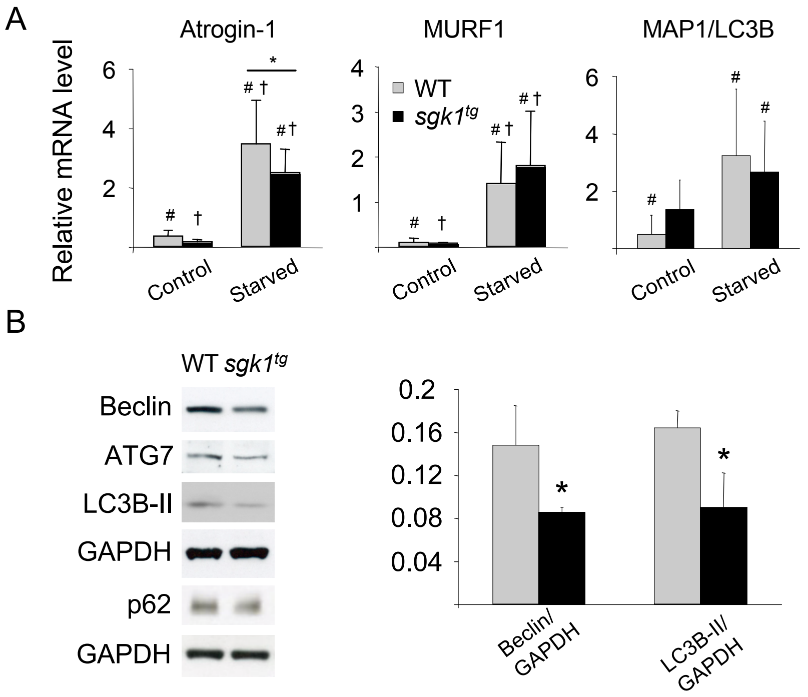

Figure-S5 (Cohn)

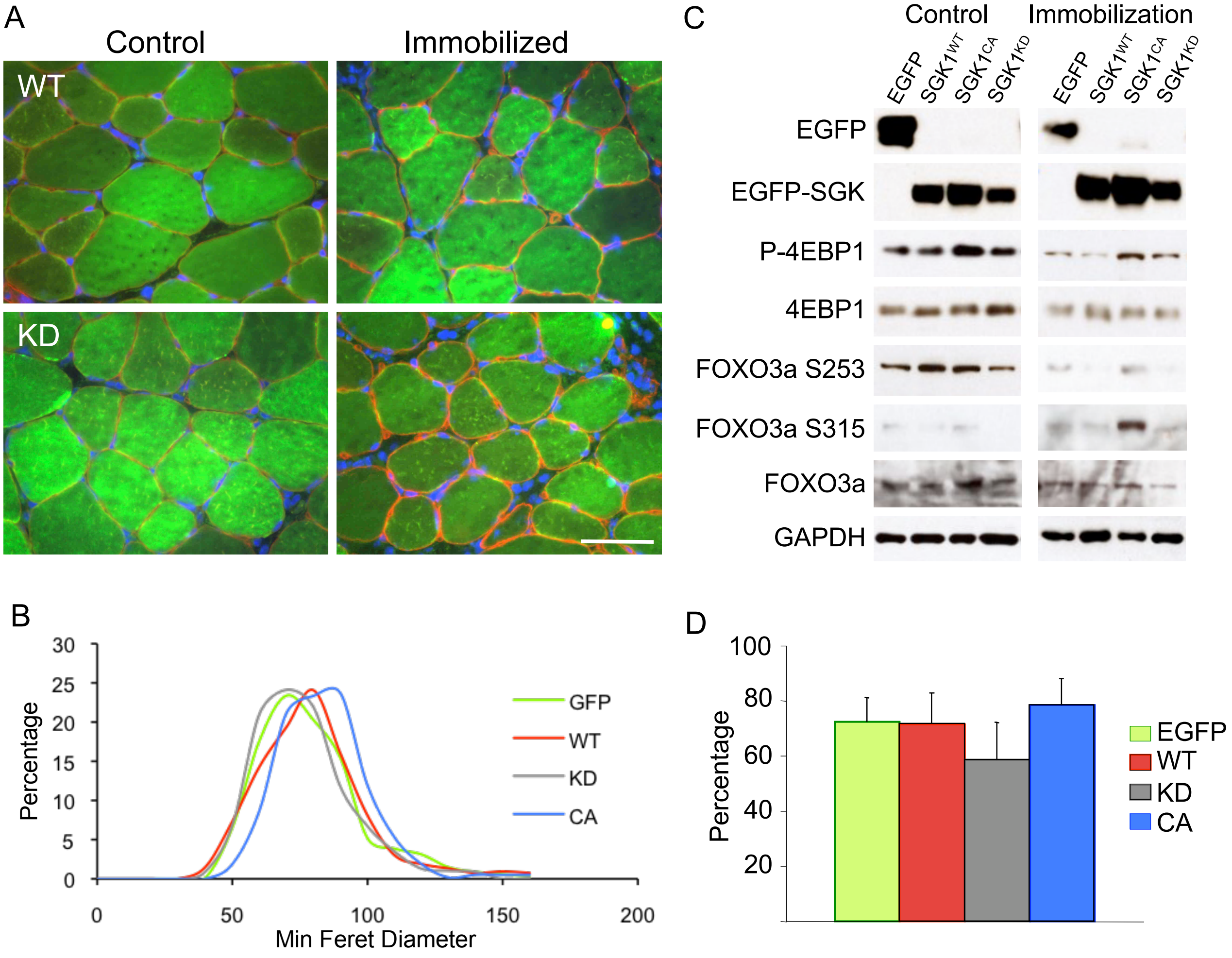

Figure-S6 (Cohn)
